# Comparative Analysis of Epidermal Differentiation Genes of Crocodilians Suggests New Models for the Evolutionary Origin of Avian Feather Proteins

**DOI:** 10.1093/gbe/evy035

**Published:** 2018-02-12

**Authors:** Karin Brigit Holthaus, Bettina Strasser, Julia Lachner, Supawadee Sukseree, Wolfgang Sipos, Anton Weissenbacher, Erwin Tschachler, Lorenzo Alibardi, Leopold Eckhart

**Affiliations:** 1 Research Division of Biology and Pathobiology of the Skin, Department of Dermatology, Medical University of Vienna, Austria; 2 Dipartimento di Scienze Biologiche, Geologiche ed Ambientali (BiGeA), University of Bologna, Italy; 3 Clinical Department for Farm Animals and Herd Management, University of Veterinary Medicine Vienna, Austria; 4 Vienna Zoo, Vienna, Austria

**Keywords:** crocodiles, alligators, comparative genomics, integument, skin, feathers

## Abstract

The epidermis of amniotes forms a protective barrier against the environment and the differentiation program of keratinocytes, the main cell type in the epidermis, has undergone specific alterations in the course of adaptation of amniotes to a broad variety of environments and lifestyles. The epidermal differentiation complex (EDC) is a cluster of genes expressed at late stages of keratinocyte differentiation in both sauropsids and mammals. In the present study, we identified and analyzed the crocodilian equivalent of the EDC. The gene complement of the EDC of both the American alligator and the saltwater crocodile were determined by comparative genomics, de novo gene prediction and identification of EDC transcripts in published transcriptome data. We found that crocodilians have an organization of the EDC similar to that of their closest living relatives, the birds, with which they form the clade Archosauria. Notable differences include the specific expansion of a subfamily of EDC genes in crocodilians and the loss of distinct ancestral EDC genes in birds. Identification and comparative analysis of crocodilian orthologs of avian feather proteins suggest that the latter evolved by cooption and sequence modification of ancestral EDC genes, and that the amplification of an internal highly cysteine-enriched amino acid sequence motif gave rise to the feather component epidermal differentiation cysteine-rich protein in the avian lineage. Thus, sequence diversification of EDC genes contributed to the evolutionary divergence of the crocodilian and avian integuments.

## Introduction

Crocodilians are a clade of semiaquatic, predatory reptiles comprising 24 species of which 14 belong to the family Crocodylidae, 8 to Alligatoridae, and 2 to Gavialidae (Li et al. 2007; http://www.reptile-database.org; last accessed September 30, 2017). The phylogenetically closest extant relatives of crocodilians are the birds. These two groups constitute the clade Archosauria and their last common ancestor lived 219–255 Ma (Shen et al. 2011; Chiari et al. 2012). After the evolutionary split between crocodilians and birds, the phenotypes of their integuments have diverged significantly. Birds have evolved feathers and beaks and only the legs are covered with scales, whereas crocodilians have an “armored” skin consisting of epidermal scales, in many cases located on top of dermal bony plates (osteoderms). In contrast to the body scales which typically develop from placodes (Musser et al. 2015; Di-Poï and Milinkovitch 2016), recent studies have indicated that the scales present on the head of crocodilians are formed by physical cracking (Milinkovitch et al. 2013).

**Fig. 1.**
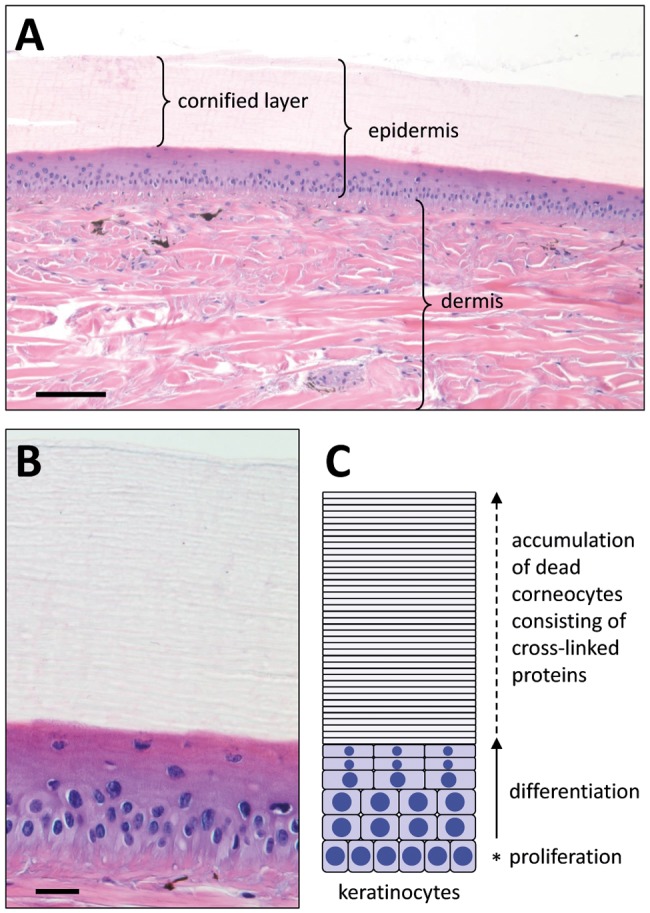
—Terminally differentiated epidermal keratinocytes form the barrier to the environment in crocodiles. Thin sections of the skin of a crocodile (*Crocodylus moreletii*) were stained with hematoxylin and eosin (*A* and *B*). Scale bars: 100 µm (*A*), 20 µm (*B*). Epidermal keratinocyte differentiation is depicted schematically (*C*). Epidermal differentiation is characterized by the expression of specific differentiation-associated proteins many of which are encoded in the epidermal differentiation complex (EDC). The final step of differentiation is cornification during which the nucleus is degraded and structural proteins are cross-linked.

In histology, crocodile scales display several layers of living keratinocytes and a thick cornified layer (fig. 1). The latter comprises multiple sublayers of cornified, enucleate keratinocytes and resembles the stratum corneum of mammalian epidermis, although the stratum corneum of crocodiles is generally much thicker and remains relatively compact during histological sectioning (Alibardi 2011) (fig. 1). In the terminology of herpetological skin research, the “hard” outer compartment of scales is referred to as the beta-layer, and the inner compartment of scales and the “soft” hinge regions localized between scales are called alpha-layer of the epidermis (Baden and Maderson 1970; Maderson 1985; Landmann 1986; Alibardi 2005, 2011; Alibardi and Toni 2006). The quantitatively predominant proteins of the reptilian beta-layer and of epidermal appendages such as claws are the corneous beta proteins (CBPs), traditionally called beta-keratins (Gregg and Rogers 1986; Presland et al. 1989; Knapp et al. 1993; Alibardi and Toni 2006; Toni et al. 2007; Greenwold and Sawyer 2010). CBPs are conserved in reptiles and birds but are absent in mammals. Apart from the presence of these proteins, very little is known about the molecular composition of the epidermal layers in crocodilians.

The sequencing of genomes of representatives from all major clades of amniotes has facilitated a major advance in the elucidation of genes that determine the structure and function of the epidermis in mammals, reptiles and birds. In particular, it has become clear that a gene cluster originally defined as the Epidermal Differentiation Complex (EDC) in mammals is shared, with clade-specific modifications, among all amniotes investigated so far. The organization of the EDC has been previously defined for birds, lizards, snakes, and turtles (Strasser et al. 2014, 2015; Holthaus et al. 2016, 2017). EDC genes were named according to a previously established nomenclature system (Strasser et al. 2014), in which gene names begin with “Epidermal Differentiation (ED)” and, in the second part, describe either the amino acid composition or the presence of particular amino acid sequence motifs of the encoded proteins.

Several individual genes within the EDC of sauropsids are expressed in differentiated epidermal keratinocytes, for example, loricrin (*LOR*) in lizard scales (Strasser et al. 2014), scaffoldin (*SCFN*) in avian claws and feathers (Strasser et al. 2015), Epidermal Differentiation Cysteine-Rich Protein (*EDCRP*) in feathers (Strasser et al. 2015) and Epidermal Differentiation Protein starting with a MTF motif and rich in Histidine (*EDMTFH*) in feathers (Alibardi et al. 2016). Moreover, there is accumulating evidence for an evolutionary origin of CBP genes within an ancestral EDC of sauropsids (Strasser et al. 2014). CBP genes are present in the EDCs of all sauropsids investigated so far, with additional CBP genes of birds and turtles being present at loci outside of the EDC as a consequence of gene amplification and translocation events (Ng et al. 2014; Holthaus et al. 2016). In crocodilians, CBPs have been characterized at the gene and protein levels (Sawyer et al. 2000, 2003; Alibardi and Toni 2007; Toni et al. 2007; Dalla Valle et al. 2009; Ye et al. 2010; Greenwold and Sawyer 2013). Immuno-crossreactivity studies have suggested that crocodilian homologs of avian feather CBPs are expressed in the subperiderm, an embryonic layer of the epidermis that is shared only between crocodiles and birds (Alibardi and Thompson 2002; Sawyer et al. 2003; Alibardi et al. 2006), suggesting that there are evolutionary-developmental links between the subperiderm and feathers (Sawyer and Knapp 2003; Sawyer et al. 2003, 2005; Alibardi et al. 2006). For crocodilians, only CBP genes and a few isolated EDC genes, identified by single gene BLAST searches (Mlitz et al. 2014), have been described so far.

In this study, we have identified the entire sets of EDC genes present in 2 species of crocodilians, and we have compared the organization of the crocodilian EDC with that of birds. This study has implications on the genetic control of cell differentiation in crocodilian epidermis and on the evolutionary origin of feather genes in birds.

## Materials and Methods

### Genome Sequences and Gene Identification

Genes were predicted in the genome sequences of the following two crocodilian species (St John et al. 2012; Green et al. 2014): the American alligator (*Alligator mississippiensis*) and the saltwater crocodile (*Crocodylus porosus*). Accession numbers of genome sequence scaffolds corresponding to the EDC can be found in supplementary tables S2 and S3, Supplementary Material online. To predict the coding sequences of EDC genes, the amino acid sequences of EDC proteins of chicken (*Gallus gallus*) and turtle (*Chrysemys picta bellii*), already identified in previous studies (Strasser et al. 2014; Holthaus et al. 2016), were used as queries. These queries were then used in tBLASTn searches against the nucleotide sequence positioned between the genes (*S100A12* and *S100A11*) bordering the EDC in the investigated species. Genes coding for functional proteins were included in comparative studies whereas pseudogenes belonging to EDC gene families with functional members in the same species were not investigated further. In case of EDC regions with seemingly low gene density, the nucleotide sequence of the region in question was translated in silico and additional open reading frames of candidate EDC genes were identified by our already published protocol (Strasser et al. 2014). In the NCBI browser for “genomic regions, transcripts, and products” (https://www.ncbi.nlm.nih.gov/gene/; last accessed September 29, 2017) information about exon coverage by RNA-seq reads was consulted to check for transcribed regions in the EDC of *A. mississippiensis*. Nucleotide sequences of transcribed regions were translated in all reading frames and the possible translation products were compared with known EDC proteins of other amniotes. To test for expression of predicted crocodilian EDC genes, we performed tBLASTn searches in the transcriptome of *A. mississippiensis* (St John et al. 2012; Green et al. 2014). The default parameters for tBLASTn searches at the NCBI browser were used, whereby the filter for low complexity regions in sequences was deactivated.

### Bioinformatic Analysis of Amino Acid Sequences Encoded by EDC Genes

Amino acid sequences were aligned using the program MultAlin (Corpet 1988). The ProtParam software tool at the ExPASy portal (Artimo et al. 2012) was used to calculate amino acid percentages.

### Animal Tissue and Histology

Morelet’s crocodile (*Crocodylus moreletii*) was kept at the Vienna Zoo, Vienna, Austria as part of an international breeding program. Skin tissue was sampled through a biopsy at the flank of a 3-year-old female Morelet’s crocodile in agreement with the national laws regulating animal welfare and the guidelines of Good Veterinary Practice. For histological investigation, the sample was fixed with 7.5% formaldehyde, embedded in paraffin, thin-sectioned and stained with hematoxylin and eosin according to a published protocol (Mlitz et al. 2014).

### RT-PCR

A part of the scaled skin tissue of *C. moreletii* was used to prepare RNA according to a published protocol (Mlitz et al. 2014; Strasser et al. 2014). The RNA was reverse-transcribed to cDNA which was subsequently amplified by PCRs with primers designed in *C. porosus* for genes of the EDC (supplementary table S4, Supplementary Material online). The PCR products were purified and sequenced. The nucleotide sequences of cDNAs were submitted to GenBank (Accession numbers MG243696, MG243697, MG243698).

## Results

### Identification of the Epidermal Differentiation Complex in Crocodilians

**Fig. 2.**
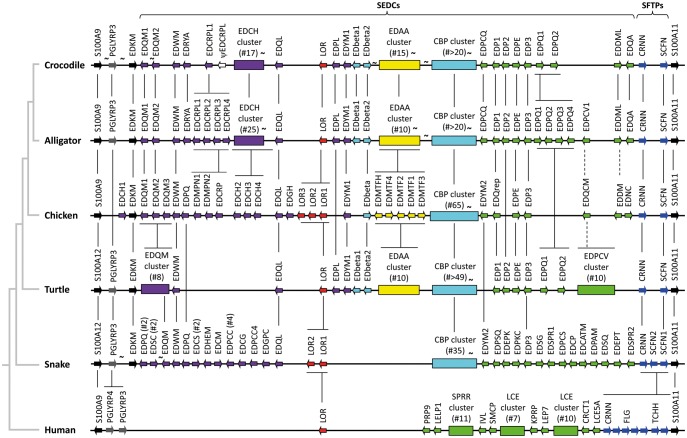
—Structure of the epidermal differentiation complex (EDC) in crocodilians. The genes of the EDC complex of the saltwater crocodile (*Crocodylus porosus*) and the American alligator (*Alligator mississippiensis*) are compared with those of a bird (chicken, *Gallus gallus*), a turtle (*Chrysemys picta*), a snake (representing squamate reptiles, Burmese python, *Python bivittatus*), and humans. A schematic depiction of the locus is given with the orientation of genes indicated by arrows. Simple EDC (SEDC) genes containing a single coding exon are shown as colored arrows with a black frame while other genes are shown as filled arrows. Gene clusters of more than four members are represented as boxes over which the number of genes is indicated after the symbol #. In the EDC of the snake, arrows labeled by # represent groups of genes. Orthology is shown by black vertical lines connecting genes or gene families. Discontinuity in the genome assembly, where the EDC was tentatively reconstructed through interspecies comparison, is indicated with the symbol ∼. Note that the schemes are not drawn to scale. EDbeta genes belong to the corneous beta protein (CBP)/beta-keratin genes. In this schematic, the names of the chicken genes *EDSC* and *EDCH5* (Strasser et al. 2014) were changed to *EDQM3* and *EDPQ*, respectively, to indicate orthologies. CBP, corneous beta-protein (also known as beta-keratin); ψ, pseudogene; SFTP, S100 fused-type protein.

To identify the EDC gene complement of the American alligator (*Alligator mississippiensis*) and the saltwater crocodile (*Crocodylus porosus*), sequences of EDC-encoded proteins of chicken and turtle (*Chrysemys picta bellii*) were used as queries in tBLASTn searches of the crocodilian genomes (St John et al. 2012; Green et al. 2014) with a focus on the region flanked by S100A genes (fig. 2, see below for a detailed description of organization and gene content of the crocodilian EDC). In addition, EDC genes within the latter region were predicted de novo according to the approach described for other sauropsids (Strasser et al. 2014; Holthaus et al. 2016, 2017). The existence of RNA-sequencing (RNA-seq) reads matching EDC genes of the American alligator (supplementary table S2, Supplementary Material online) validated most of the predictions. Crocodilian EDC genes were named according to the previously established nomenclature system (Strasser et al. 2014). Full names are listed in supplementary table S1, Supplementary Material online, whereas only abbreviated names are used in the text to simplify reading.

**Fig. 3.**
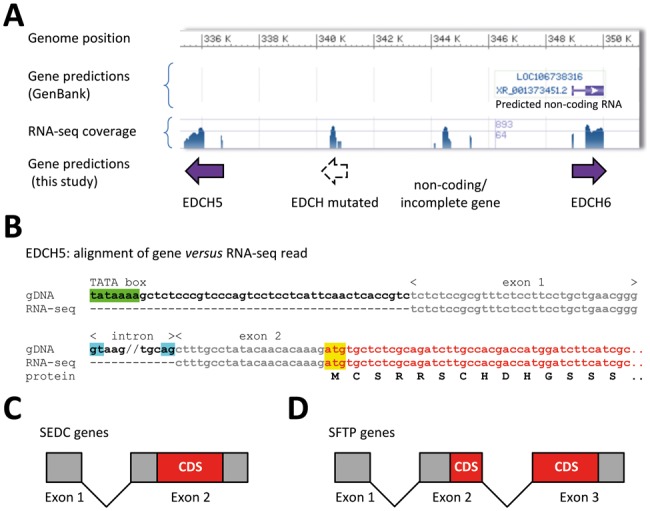
—Identification of genes in the EDC of crocodilians. (*A*) Representative view (GenBank accession number NW_017707560.1) in the genomic data browser (NCBI GenBank) for *Alligator mississippiensis* indicating annotations, RNA-seq coverage (blue peaks), and predictions made in this study. (*B*) Alignment of the nucleotide sequence of the *EDCH5* gene predicted in our study with a RNA-seq read from the sequence read archive (SRA) (accession number SRR3208124.3838205.1, determined in experiment SRX1616862). Nucleotides of coding and noncoding regions are indicated by red and gray fonts, respectively. The TATA box is highlighted in green, the splice sites in blue and the start of translation in yellow. The amino acid sequence of the translated product is shown underneath the nucleotide sequence. The analysis of *EDCH6*, corresponding to *LOC106738316* in panel A, is shown in supplementary figure S3, Supplementary Material online. (*C* and *D*) Schematic depiction of the exon–intron structures and the positions of coding sequences (CDS) in SEDC (simple EDC) and S100 fused-type protein (SFTP) genes.

When comparing our EDC predictions with the current annotation of the crocodilian genome assemblies (October 2017), we found that some EDC genes were correctly annotated, whereas others were missed by the automatic algorithms and some gene annotations included splice sites and reading frames that were not plausible (fig. 3*A*). Apparently, the short open reading frames and the low sequence complexity of EDC genes did not allow the automatic algorithm to identify the coding regions of many crocodilian EDC genes. By contrast, our approach facilitated predictions of both Simple EDC (SEDC) genes in which the coding region is confined to one exon (fig. 3*C*) and S100 fused-type protein (SFTP) genes in which the coding sequence is present on 2 exons (fig. 3*D*) (supplementary figs. S1–S3 and tables S2 and S3, Supplementary Material online). A single 5′-noncoding exon characteristic for both SEDC (supplementary fig. S3, Supplementary Material online) and SFTP genes was identified for some but not all genes based on intron spanning RNA-seq reads (fig. 3*B* and supplementary table S2, Supplementary Material online). The relative arrangement of crocodilian genome sequence scaffolds containing EDC genes (supplementary tables S2 and S3, Supplementary Material online) was predicted by alignment to the orthologous regions of the EDC in other amniotes (fig. 2).

### The EDC of Crocodilians Is Syntenic with the EDCs of Birds and Turtles

The overall organization of the crocodilian EDC is syntenic with the EDC organization of birds (Archosauria) and also with that of the EDC in turtles (fig. 2). Only 2 SFTP genes (*CRNN* and *SCFN*) are present close to one end of the EDC, and SEDC genes form the main part of the EDC in crocodilians. Comparative analysis suggests that a cluster of crocodilian CBP (beta-keratin) genes is syntenic with CBPs in the EDC of other sauropsids (fig. 2). Two EDC genes, *EDCH* and *EDDM*, that were previously identified in birds but not in other sauropsids, have orthologs in crocodilians (fig. 2), suggesting an evolutionary origin in an ancestral archosaur. Some other features of the EDC, such as the presence of genes encoding proline-rich proteins, are shared between crocodilians, birds, turtles, and squamates whereas other genes are unique to the crocodilians (see fig. 2 for details). Yet other genes, such as *EDKM, EDWM, EDQL, EDYM1, EDP3*, and *SCFN*, were confirmed to be conserved in all major sauropsid clades, and *PGLYRP3*, *LOR*, and *CRNN* of crocodilians have orthologs in mammals (Strasser et al. 2014; Holthaus et al. 2016, 2017) (fig. 2).

### RNA-Seq Data Suggest That EDC Genes Are Expressed in the Skin but Not in Internal Organs

**Fig. 4.**
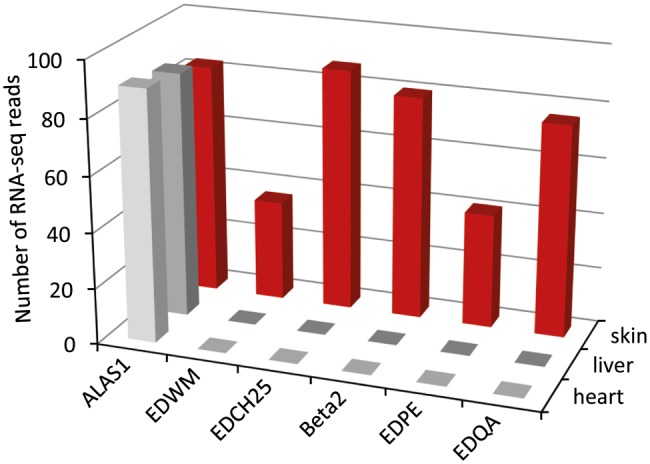
—RNA sequencing data suggest skin-specific expression of EDC genes in the alligator. To determine the tissue expression pattern of representative EDC genes of *Alligator mississippiensis* in a semiquantitative manner, we screened the RNA-seq data deposited in the GenBank sequence read archive (SRA). The whole amino acid sequences of the selected EDC proteins were used as queries in tBLASTn searches. Transcriptome data from juvenile belly skin, liver, and heart (St John et al. 2012) were investigated. The RNA-seq reads yielding 100% identical matches to the query sequence were counted and plotted on the graph. Transcripts of the ubiquitously expressed gene *5′-aminolevulinate synthase 1* (*Alas1*) were counted as a positive control for the analysis of each transcriptome. Accession numbers of transcriptome data: SRX1616878 (juvenile heart), SRX1616880 (juvenile liver), and SRX1616862 (juvenile belly skin).

To explore the tissue expression pattern of crocodilian EDC genes, we compared the number of RNA-seq reads corresponding to specific EDC genes in the tissue transcriptomes of the American alligator (St John et al. 2012). BLAST hits matching to *EDWM*, *EDCH25*, *Beta2* (corneous beta-protein 2), *EDPE*, and *EDQA* (for full names, see supplementary table S1, Supplementary Material online) were obtained at high numbers in the skin but not in the heart or liver (fig. 4). The housekeeping gene *ALAS1* (Beer et al. 2015), used as a control, showed a similar level of expression in all three tissues (fig. 4).

### Proteins Encoded by Crocodilian EDC Genes Are Enriched for a Small Set of Amino Acids and Sequence Repeats

**Fig. 5.**
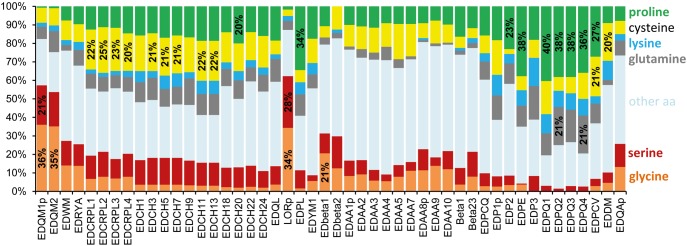
—Proteins encoded by EDC genes of the American alligator are enriched for a subset of amino acid residues. The amino acid (aa) compositions (% of total aa residues) of SEDC proteins of the *Alligator mississippiensis* are shown. The order of the protein data corresponds to the order of their respective genes on the EDC (fig. 2). Note that not all the translation products of the two large gene clusters, that is, corneous beta proteins (CBP) and EDCH, are included here: Data for the proteins encoded by the first and the last gene of the CBP cluster and 11 out of 25 EDCH proteins are shown. The letter “p” at the end of protein names indicates that only partial sequences were available for analysis.

Similar to their orthologs in other amniotes, EDC proteins of crocodilians contain high amounts of one or more of the amino acids glycine (G), serine (S), proline (P), lysine (K), cysteine (C), and glutamine (Q) (fig. 5). In many EDC proteins, the percentage of one of these amino acids exceeds 20%, as is the case for glycine in EDQM1-2, loricrin and EDbeta1; serine in EDQM1 and loricrin; cysteine in EDCRPL1-3, EDPCV and several EDCH proteins; proline in EDPL, EDP2, EDPE, EDPQ1-4 and EDPCV, and glutamine in EDPQ2 and 4. Particularly, striking are the proline contents ∼40% in EDPQ proteins (fig. 5).

Many EDC proteins of crocodilians exhibit sequence repeats rich in the above-mentioned amino acids (supplementary fig. S4, Supplementary Material online). Another characteristic of EDC proteins of crocodilians and other amniotes are amino- and carboxy-terminal sequence motifs containing lysine and glutamine (supplementary fig. S5, Supplementary Material online), corresponding to sites of Nɛ-(γ-glutamyl)lysin protein cross-linking via transglutamination in mammalian EDC proteins (Candi et al. 2005). Thus, the amino acid sequences of EDC proteins of crocodilians are compatible with roles as structural components of cornified keratinocytes.

### Comparative Analysis of Crocodilian and Avian EDCs Suggests Lineage-Specific Gene Alterations

**Fig. 6.**
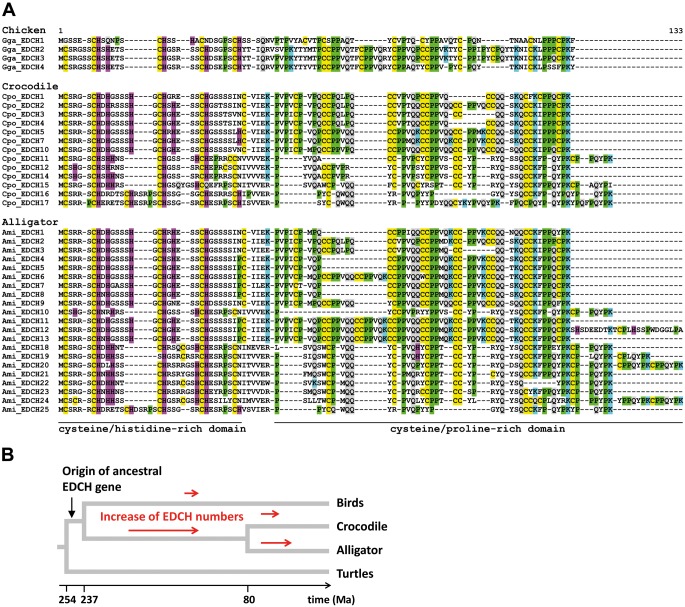
—The number of EDCH proteins has expanded in crocodilians. (*A*) Amino acid sequences of EDCH (Epidermal Differentiation protein containing cysteine histidine motifs) proteins were aligned based on the translation of *EDCH* genes identified in the EDCs of the chicken, the American alligator, and the saltwater crocodile. Histidine is highlighted in magenta, other colors are as indicated in figure 5. (*B*) Simplified model of EDCH evolution. The schematic diagram depicts a hypothesis about the change in the number of EDCH genes over time. Specific rates of gene duplication and pseudogenization were not estimated. TimeTree estimates of evolutionary divergence times are indicated (Kumar et al. 2017). Mya, million years ago.

Epidermal Differentiation proteins containing Cysteine Histidine motifs (EDCHs), which have a unique organization with an amino-terminal domain rich in cysteine and histidine residues and a carboxy-terminal domain rich in cysteine and proline residues (fig. 6*A*), were previously identified in birds while no orthologs for these proteins were found in turtles and lepidosaurs (Strasser et al. 2014; Holthaus et al. 2016, 2017). In the present study, we identified EDCH genes in both the alligator and crocodile EDC (fig. 2). The expression of a representative EDCH gene (EDCH25) in the skin of the American alligator was confirmed by transcriptome data (fig. 4). By reverse-transcriptase polymerase chain reaction (RT-PCR), we could also detect the expression of an EDCH gene (presumably orthologous to EDCH10 of the saltwater crocodile) in the scale skin of Morelet’s crocodile (Belize crocodile) (*C. moreletii*) (supplementary table S4 and fig. S6, Supplementary Material online). Interestingly, 17–25 EDCH genes are present in crocodilians with at least 13 (*C. porosus*) and 21 (*A. mississippiensis*) having an intact and complete open reading frame (supplementary figs. S1 and S2, Supplementary Material online) whereas only three to five copies are present in birds. Considering the lengths of time between the divergence of the evolutionary lineages (fig. 6*B*), it appears likely that the number of EDCH genes was low in the last common ancestor of Archosauria and increased specifically during the evolution of crocodilians.

**Fig. 7.**
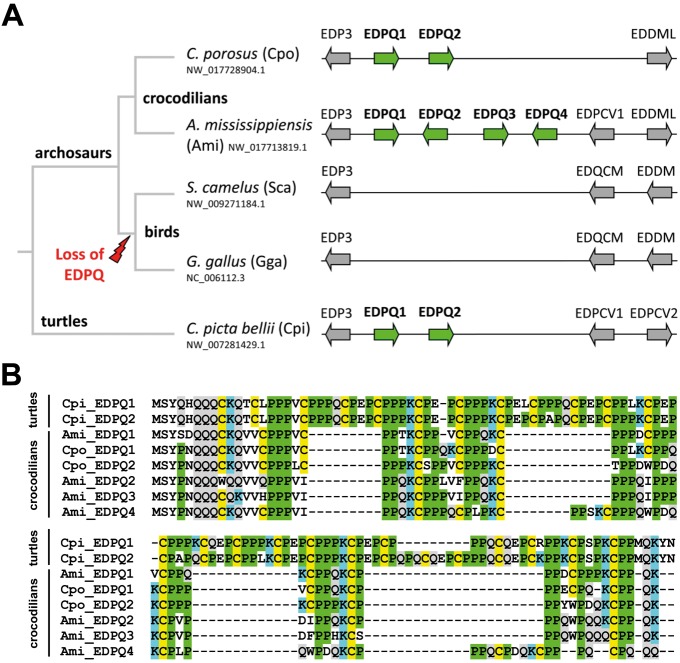
—EDPQ is conserved in crocodilians but it has been lost in birds. (*A*) Schematic phylogenetic tree of the EDPQ locus in two representive bird and crocodilian species and turtles as an outgroup. Conservation of flanking genes (*EDP3*, *EDPCV1*/*EDQCM*, and *EDDM*) confirmed correct identification of the locus of species indicated. (*B*) Alignment of turtle and crocodile EDPQ amino acid sequences. The abbreviations of species names are shown in panel (*A*).

Two and four *EDPQ* (*Epidermal Differentiation proteins rich in proline [P] and glutamine [Q]*) genes were found in the EDCs of the saltwater crocodile and the American alligator, respectively. In a previous study, two *EDPQ* genes were also identified in the turtle *C. picta* (Holthaus et al. 2016). By contrast, representative bird species such as chicken and ostrich do not have *EDPQ* genes (fig. 7). These data suggest that the primordial *EDPQ* gene originated in a common ancestor of turtles and archosaurs but was later lost in the bird lineage (fig. 7). Likewise, the ancestral *EDP2* gene appeared to have undergone inactivation in birds (supplementary fig. S7, Supplementary Material online).

### The Crocodilian EDC Comprises Orthologs of Avian Feather Protein Genes

The EDC of the chicken comprises at least three types of genes that encode feather proteins: feather-CBPs (also called beta-keratins) (Greenwold and Sawyer 2010; Ng et al. 2014; Wu et al. 2015), EDMTFH (also called histidine-rich protein, HRP) (Strasser et al. 2014; Alibardi et al. 2016), and EDCRP (Strasser et al. 2014, 2015). Previous studies have shown that feather-CBPs evolved after the divergence of the avian from the crocodilian lineage (Dalla Valle et al. 2009; Greenwold and Sawyer 2011; Greenwold and Sawyer 2013). The characterization of crocodilian EDC genes allowed us to study the origin of the two other known feather proteins.

Chicken EDMTFH is characterized by a high histidine content which is not conserved in orthologous proteins of numerous other birds (Alibardi et al. 2016). EDMTFH belongs to the avian EDMTF proteins which have amino acid sequences similar to those of crocodilian EDAA proteins (supplementary fig. S8, Supplementary Material online). The chicken EDMTF genes form a subcluster within the EDC sharing synteny with the *EDAA* clusters of turtles and crocodilians (fig. 2). Thus, the apparent homology of avian EDMTFs, including the feather protein EDMTFH (Alibardi et al. 2016), and crocodilian EDAAs suggests that, in the avian lineage, one or more ancestral *EDAA* genes were coopted for functions in feathers.

**Fig. 8.**
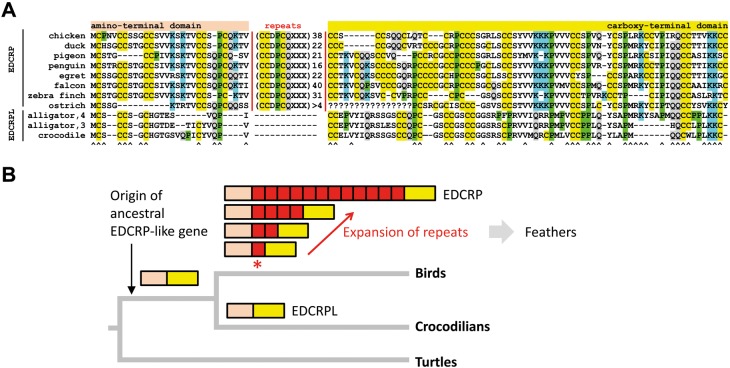
—The avian feather protein EDCRP has evolved by the origin and expansion of an internal sequence repeat. (*A*) The amino acid sequences of avian Epidermal Differentiation Cysteine-Rich Protein (EDCRP) and crocodilian EDCRP-like (EDCRPL) proteins were aligned. An internal sequence repeat is present multiple times (as indicated) with variable carboxy-terminal amino acid residues (indicated by X) in birds, whereas it is absent in EDCRPLs of crocodiles. The ^ symbols below the sequence alignment indicate positions of amino acid residues that were conserved in one or more representatives of both crocodilians and birds. Species names and the number of the respective EDCRPL isoform of the alligator are indicated in front of the sequences. EDCRP sequences of birds were published previously (Strasser et al. 2015). Species: chicken (*Gallus gallus*), duck (*Anas platyrhynchos*), pigeon (*Columba livia*), penguin (*Pygoscelis adeliae*), egret (*Egretta garzetta*), falcon (*Falco cherrug*), zebra finch (*Taeniopygia guttata*), ostrich (*Struthio camelus*), alligator (*Alligator mississippiensis*), and crocodile (*Crocodylus porosus*). (*B*) Schematic model of EDCRP evolution. The presence and domain organization of EDCRP-like proteins were mapped onto a schematic phylogenetic tree of archosaurs with turtles as outgroup. The origin of the internal sequence repeat motif is indicated by an asterisk. EDCRP is a component of feathers in modern birds.

Shared synteny and high similarity of amino acid sequences in the amino- and carboxy-terminal domains of the encoded proteins identify *EDCRP-*like genes in crocodilians as orthologs of avian *EDCRP* (fig. 8). The orthologous EDCRP-type proteins of crocodilians and birds differ by the absence or presence of the repeated sequence motif CCDPCQ(K/-)(T/P)(V/-), whereby the dash indicates that there are repeats lacking this amino acid sequence position (fig. 8). This sequence is absent in crocodilian EDCRP-like proteins but present and amplified (with some deviations in its carboxy-terminal residues, indicated by X in the alignment in fig. 8*A*) up to >50-fold in the central part of EDCRPs of different clades of birds (Strasser et al. 2015) (fig. 8*B*). We conclude that the evolution of the feather protein EDCRP represents an intramolecular structural innovation that has occurred in the avian lineage after its divergence from the crocodilian lineage.

## Discussion

### Implications on Epidermal Differentiation in Archosaurs

The results of the present study show, for the first time, the presence of an EDC in crocodilians and, by comparing its organization with that of other amniotes, help to build a model for the evolution of epidermal differentiation in early archosaurs. Crocodilians are the last major clade of amniotes (Strasser et al. 2014; Holthaus et al. 2016, 2017) for which the structure of the EDC is reported. Therefore, the results of the present study complete the draft inventory of diversified EDC structures of amniotes.

Previous studies of crocodilian epidermal proteins had focused primarily on highly abundant CBPs/beta-keratins (Sawyer et al. 2000; Alibardi and Toni 2007; Toni et al. 2007; Dalla Valle et al. 2009; Ye et al. 2010; Greenwold and Sawyer 2013). In agreement with previous gene (Greenwold and Sawyer, 2013) and proteome (Alibardi and Toni 2007; Toni et al. 2007) analyses, we conclude that the number of CBP genes in crocodilians is the lowest among reptiles. We identified 22 complete CBP genes (including EDbetas) in *A. mississippiensis* and *C. porosus* and 6 partial CBP genes in the *C. porosus* genome (supplementary figs. S1*B* and S2*B*, Supplementary Material online), whereas lepidosaurs have at least 35 CBPs (Holthaus et al. 2017), and turtles have >70 CBPs (Li et al. 2013; Holthaus et al. 2016). Perhaps, the relatively small number of CBPs is related to the limited variety of types and textures of the scales in crocodilians (Pough et al. 2001). Comparative studies of other epidermis-associated genes yielded only limited information on crocodilians (Dalla Valle et al. 2011; Abbas Zadeh et al. 2017). Thus, the identification of multiple crocodilian EDC genes represents a significant advancement in the characterization of epidermal differentiation in crocodilians.

Based on shared synteny, we propose a draft organization of the EDC in the American alligator and the saltwater crocodile. There is a high degree of similarity in the EDCs of both species, suggesting that many features have been inherited from their last common ancestor and therefore from the last common ancestor of all crocodilians. In line with this notion, a preliminary investigation of the genome of the Indian gharial (*Gavialis gangeticus*) (Green et al. 2014) indicated conservation of amino acid sequences encoded by EDC genes and syntenic organization of the EDC (supplementary fig. S9, Supplementary Material online, and data not shown). The current genome assemblies of crocodilians are compatible with a continuous EDC like in the human genome. However, due to gaps in these sequence assemblies, especially in the genome of the American alligator, discontinuities in the arrangement of EDC genes can also not be fully excluded. A rearrangement of the EDC was reported for the opossum (Vanhoutteghem et al. 2008), and discrepancies exist between the chicken EDC models in the Gallus_gallus-4.0 assembly (Bellott et al. 2010; Strasser et al. 2014; Holthaus et al. 2016) which was used in the present study, and the Gallus_gallus-5.0 assembly (Warren et al. 2017). As the expression of EDC genes may depend on their location in topologically associating domains (TADs) (Poterlowicz et al. 2017), further detailed characterization of the EDC gene loci in crocodilians and other amniotes may yield important insights into the regulation of gene expression during epidermal differentiation.

Comparison of alligator and crocodile versus chicken shows that there is a high degree of conservation of EDC genes in archosaurs. Only few SEDC genes of crocodilians lack an ortholog in birds and *vice versa*. However, there are also important differences which have likely contributed to the divergent evolution of skin phenotypes in crocodilians and birds. In fact, the epidermal stratum corneum of crocodilians evolved into a particularly mechano-resistant and water-proof component of the skin, and the beak and particularly the feathers of birds represent unique evolutionary innovations that depended on modifications of the epidermal differentiation process.

### Divergent Evolution of Crocodilian and Avian EDC Genes

Our data suggest that the EDC underwent only few changes in gene composition and arrangement in the crocodilian lineage after its split from the avian lineage. These changes were inferred from our comparison of the EDCs of crocodilians with the EDC of birds and turtles. Both the saltwater crocodile and the American alligator have at least 4 times as many EDCH genes as birds, suggesting that the EDCH gene cluster expanded in the stem lineage of crocodilians (fig. 6). Other alterations of the EDC affected single genes (leading to different numbers of EDCRPL and EDPQ genes in the saltwater crocodile vs. the American alligator, fig. 2) and have likely occurred after the divergence of sublineages within crocodilians (Pough et al. 2001).

Importantly, the results of this study also suggest a scenario for the evolution of EDC genes in the stem lineage of birds. We put forward the hypothesis that the evolution of feather proteins involved the cooption of genes encoding CBP/beta-keratins, EDAAs (termed EDMTFs in birds), and a precursor of EDCRP. The cooption of epidermal structural proteins may be a common theme in the evolution of feathers and hair (Eckhart et al. 2008; Wagner 2014). It remains to be determined which modifications in the amino acid sequences of CBP/beta-keratins and EDAAs contributed to their functions as structural proteins of feathers. For the feather gene *EDCRP*, we identified orthologs in crocodilians (figs. 2 and 8), whereas no orthologs are present in turtles (Holthaus et al. 2016). The characteristic feature of EDCRP is its uniquely high number of cysteine residues which, in analogy to the numerous cysteine residues of mammalian hair proteins, have been proposed to serve as sites of intermolecular cross-linking via disulfide bonds (Strasser et al. 2014). The crocodilian orthologs of EDCRP have a cysteine content of >20%, which is above the average cysteine content of proteins encoded within the EDC. In the evolutionary lineage leading to birds, a cysteine-rich sequence motif appeared probably by duplication and mutation of a neighboring sequence in the ancestral gene, and this sequence motif was again manifold amplified. Consequently, the length of EDCRP increased in the avian lineage, leading a much higher total number of cysteine residues per molecule than those present in its crocodilian counterparts (160 cysteine residues in chicken EDCRP vs. a maximum of 22 cysteine residues in crocodilian EDCRPLs). Proteomic analysis of cornified feathers has confirmed the integration of EDCRP into the permanent parts of feathers (Strasser et al. 2014, 2015). Thus, the intramolecular modification of an EDCRP-like protein has likely contributed, together with sequence adaptations of EDAA/EDMTF proteins and CBP/feather beta-keratins, to the evolution of the heavily cross-linked protein architecture of feathers. Remarkably, feather CBPs/beta-keratins, EDMTFH, and EDCRP of the chicken were found to be expressed not only in feathers but also in the embryonic subperiderm (Strasser et al. 2015; Alibardi et al. 2016), a layer which has a homolog in crocodilians (Alibardi and Thompson 2002; Sawyer et al. 2003). This leads us to hypothesize that the primordial expression site of feather protein precursors was the subperiderm, which is evolutionarily older than feathers. Thus, it will be very interesting to determine the expression of crocodilian EDC genes not only in adult skin but also in the epidermis of embryos.

In conclusion, the results of the present comparative genomic analysis of the EDC in archosaurs provide a basis for studying the specific roles of genes involved in epidermal differentiation in crocodilians and allow to further delineate the evolutionary divergence between the crocodilian and avian skin.

## Supplementary Material

Supplementary data are available at *Genome Biology and Evolution* online.

## Competing Financial Interests

The authors declare no competing financial interests.

## Supplementary Material

Supplementary Figures and Tables
