# Supplementary material for: Comparative Analysis of Epidermal Differentiation Genes of Crocodilians Suggests New Models for the Evolutionary Origin of Avian Feather Proteins
Source: Genome Biol Evol. 2018 Feb 12;10(2):694–704. doi: 10.1093/gbe/evy035 (PMC5827346; doi:10.1093/gbe/evy035)
Supplement: Supplementary Figures and Tables [file evy035_supp.pdf]

Supplementary Data: Supplementary Figures and Tables

Comparative analysis of epidermal differentiation genes of crocodilians suggests new models for the evolutionary origin of avian feather proteins

Karin Brigit Holthaus, Bettina Strasser, Julia Lachner, Supawadee Sukseree, Wolfgang Sipos, Anton Weissenbacher, Erwin Tschachler, Lorenzo Alibardi, Leopold Eckhart

Content

Supplementary Figures S1-S9

Supplementary Tables S1-S4

A

>Ami_CRNN
MTQLQGNIEGIVSAFNAYAKEDGGCITLSKGELRQLIQQEFADVLVKPHDLQTIDQVLQRLDVEREDRIDFDEFLVLVFQVAK
ACHKKLSPCQPSGDGQGSAAQGDASRDQAQRADQEQGQKQPDAPEQDPTRPQAPETRTAEGDLSRRHTQDPEVSGTQDPEVSQ
GDGNHEAQAAKTPEHDSIRRQGQEPEQDPSHHRAQEQDPNREGQDPQVPQQDVKHEALESGAPEQAPNRHPVLQPSVSERDLD
HSHSSASERDLDHHPTLQPNTLETDLDRHPTLQPNTLETDLDRLPTLQPSTSETDLDHHQGLETEAPEQDLNSTTGRESTHNE
AHELQAPEQESQESQPDEQDQNRHQSEEPEASEHTLHHQPQEAQPTEQDLTWETQTPRVPEGDVSRGGTPFSPALQQDRGAQQ
DPREEALTAYRPYIYQCQKPPTFPYQWLPKQ

>Ami_EDAA1_partial
MFDSLDTTEDLFYQGCWDPCYRRPYWNSCWDPCTYRRPXXXGGCYPYSSRWGRRYSYGNCWPC

>Ami_EDAA2
MFDSLDVIEDLHYPGQSDCFPHQRRRPPYTCCCYDQWGRLVWRGCCWSAPPWWCRRGSSGNGWPC

>Ami_EDAA3
MFDSLETLYDLFYQGQSDCWPPTPRRTPYTCCCYDRCGRLVWRGCCWSIPPW

>Ami_EDAA4
MFDSLHTFEDLFYLGQSDFRWPPYHQRQYLCCCYDRYECLVWQGPCCYPGPWCRR

>Ami_EDAA5
MSDSLDMIEDLYYQNPSCCWRPPCRRRCWCCCYDPCTGQLIWQGWCWCPGWRSRGRYGRCWPC

>Ami_EDAA6_partial
MFDSLDMIEDLSYQXXX

>Ami_EDAA7
MSDSLDMLEELYYQSPGCCWRPRRRRPCYCCCYDPCTGELVWEGWCCCPWWGGRGRYGRRWPC

>Ami_EDAA8_partial
MSDSLDMLEDLWYPGQSNCWXXXRRPYWNSCWDPYTYRRPSWSGCWNPCTYRKPYIYNRSYGYGGLYGAGGCFPYSTRWGRRY
SAGSCWPC

>Ami_EDAA9
MTYHQSGCDDVCYTHCSYGVLYGYHGLTACWEPWTYGRPYSYGCCNPCTYRWPNRYWEPCGYGIG

>Ami_EDAA10
MSDSLDMLENFYYPGQSNCWDPCYRRPYWNSWWDPCTYRRPSWYGCWDPCTYRRPYIYDNCYGYGGLYGLGGCYPYSSRWGRR
GSWGSCWPC

>Ami_EDCH1
MCSRRSCHDHGSSSHGCHRHESSCHGSSSSINCVIEKPVPICPMPQCCPPIQQCCPPVQKCCPPVKCCQQNQQCCKFPPQYPK

>Ami_EDCH2
MCSRRSCHDHGSSSHGCHGHESSCHGSSSSINCIIEKPVPICPVPQCCPQLPQCCVPVQPCCPPMDKCCPPVQCCQQSKQCCK
IPPPCPK

>Ami_EDCH3
MCSRRSCHDHGSSSHGCHSHESSCHGSSSSINCVIEEPVPVCPVPQCCPQLPQCCVPVQQCCPPMQCCQQTKQCCKIPPQCPK

>Ami_EDCH4
MCSRRSCHDHGSSSHGCHSHESSCHGSSSSIPCIIEKPVPICPVQPCCPPVQQCCPPVQKCCPPVKCCQQSKQCCKFPPQCPK

>Ami_EDCH5
MCSRRSCHDHGSSSHGCHGHESSCHSSSSSIPCIIEKPVPVCPVQPCCPPVQQCCPPMQKCCPPVKCCQQTKQCCKFPPQCPK

>Ami_EDCH6
MCSRRSCHDHGSSSHGCHGHESSCHGSSSSIPCIIEKPVPVCPMQPCCPPVQQCCPPVQKCCPPVQQWCPPMQKCCPPVKCCQ
QTKQCCKFPPQCPK

>Ami_EDCH7
MCSRRSCHDHGASSHGCHGHESSCHSSSSSIPCILEKPVPVCTVQPCCPPVQQCCPPVQKCCPPVKCCQQSKQCCKFPPQCPK

>Ami_EDCH8
MCSRRSCHNHGSSSHGCHGHESSCHSSSSSIPCIIEKPVPVCPVQPCCPPVQQCCPPMQKCCPPVKCCQQSKQCCKFPPQ
CPK

>Ami_EDCH9
MCSRRSCHDHGSSSHGCHGNESSCHDSSSSINCVIEKPVPICPMPQCCPPVQQYCPPVQQCCPPVQQCCPPVQCCQQSQQCCK
IPPQFPK

>Ami_EDCH10
MCSHGSCHNRHRSCHGSSSHCHESRPSCNIVVVEKPYCCPVPRYYPPVSCCYPRYQYSQCCKFPQYPKCPPQYPK

>Ami_EDCH11
MCSRRSCHDHGSSSHGCHGHESSCHSSSSSIPCIIEKPVPVCPVQPCCPPVQQCCPPVQKCCPPVQQCCPPVQKCCPPVKCCQ
QTKQCCKFPPQCPK

>Ami_EDCH12
MCSRRSCHDHGSSSHGCHGHESSCHGSSSSIPCIIEKPVPICPMQPCCPPVQQCCPPVQKCCPPVQQCCPPMQKCCPPVK
CCQQSKQCCKFPPQCPKSHSDEEDTKTCPLHSSPWDGGLPA

>Ami_EDCH13
MCSRRSCHNHGSSSHGCHGHESSCHSSSSSIPCIIEKPVPVCPVQPCCPPVQQCCPPVQKCCPPVQQCCPPMQKCCPPVKCCQ
QTKQCCKFPPQCPK

>Ami_EDCH14_partial
XXXGSSSHCHESRPSCNIVVVEKPYVQACCPVPSYCPPVSCCYPRYQYSQCCKFPQYPKCPPQYPK

>Ami_EDCH15_partial
XXXGSSSHCHESRPSCNIVVVEKPYCCPVPRYCPPVSCCYPRYQYSQCCKFPQYPKYPQYPK

>Ami_EDCH16_partial
XXXGSSSHCHESRPSCNIVVVEKPYCCPVPRYCPPVSCCYPRYQYSQCCKFPQYPKYPQYPK

>Ami_EDCH17_partial
XXXGSSSHCHESRPSCNIVVVEKPYVQACCPVPSYCPPVSCCYPRYQYSQCCKFPQYPKCPPQYPK

>Ami_EDCH18
MCSRGSCHDHHNTCHRSQCGSHCHESRPSCNINEVERLSVQSWCPVQQYCPVQHYCPPTCCYPRYQYSQCCKFPPEYPKCPPQ
YPK

>Ami_EDCH19
MCSRGSCHDHHSSSHGSRCRSRCHESRPSCNITVVERPSIQSWCPVQQYCPVQHYCPPTCCYPRYQYSQCCKFPPQYPKCPLQ
YPKCPLQYPK

>Ami_EDCH20
MCSRGSCHDLHSSCHRSRRGSHCHESRPSCNITVVERPSVQSWCPVQQYCPVQPYCPPTCCYPRYQDSQCCKFPPQYPKCPPQ
YPKCPPQYPKCPPQYPK

>Ami_EDCH21
MCSRRSCHNHHSSCHRSRYGSHCHESRPSCNITVVERPFMQSWCPVQQYCPMQRYCPPTCCYPCYQYSQCCKFPPQYPKCPPQ
YPK

>Ami_EDCH22
MCSRGSCHDHHNTCHRSRCGSHCHESRPSCNITVVEWPSVKSWCPMQQYCPVQRYCPPTCCYPRYQYSQYPKCPPQYPK

>Ami_EDCH23
MCSRGSCHNHHNSCHRSRRGSHCHEYRPSCNITVVDRPSMQSWCPMQQYCPTQRCCPPTCCSPRYQYSQCYKFPPQYPKCPPQ
YPK

>Ami_EDCH24
MCSCRSCHDHHSSCHGSRCGSHCHESILYCNIMVVERPSLLTWCPMQQYCPVQLYCPPTCCYLRYQYSQCCQCPLQYRKCPPP
YPKYPPQYPKCPPQYPK

>Ami_EDCH25
MCSRRRCHDRETSCHDSRPSCHSSGSRCHESRPSCHVSVIERPPYCQWQQYRPVQPYYPGYQYSQCCKFPQYPKYPQYPK

>Ami_EDCRPL1
MCSCCSGCHGTRSVQPICYVQPVCCEPVYIHRSSGSCCQPCGSCCGSCCRGSRACPWVVIQRRPMPVCCPPLQYSAPMQQHCS
PLKKC

>Ami_EDCRPL2
MCSCCSGCHGTETICYVQPVCCEPVYIHRSSGSCCQPCGSCCGSCCGSCCWGLRSCPQVVIQRRPMPVCCPPLQYSSPMQQCC
SPLKKC

>Ami_EDCRPL3
MCSCCSGCHGTDETICYVQPVCCEPVYIQRSLESCCQPCGSCCGSCCGGSRSCPRVVIQRRPMPVCCPPLQYSAPMHQCCLPL
KKC

>Ami_EDCRPL4
MCSCCSGCHGTESVQPICCEPVYIQRSSGSCCQPCGSCCGSCCGGSRPFPRVVIQRRPMPVCCPPLQYSAPMRKYSAPMQQCC
PPLKKC

>Ami_EDDML
MAFPNQQQYKQPCLPPLVCIQKCPPRCVDQCDAACVKKHTDLHGNICAKSCTTKCVDSCDGISTMLCMTKCMDPCGAACVKEC
TTKCMCPSNTVCAKPCVTKYVDPCGTSCVMSCATPCLEPCNTICVKECVTKCMDPCGTFCAEPYVTKYGDPGCSNSAKPCITK
CVDSCNTVCVKECTTKCVDLCSTRYAKPCVTNCVDSCGTGCGKLCVTKCVDPCGTGCGKLCVTKCMDPCGTMCTKPCLTMCMN
PCSTRCAKPSVTKCVNLCGTMCGKPCITKHEDPCGTICVKECTTKCMDPCGIICTKPCVTKCVDPCTTSCVTSCVTKSTESCS
TVCIKKCTVKCVDTCSTICAKPFVPKCTDPCCPRCTASSGTTSMDPCAPVCKKTYPLQIVDLRLSKCPPVQQCCQKPKQC

>Ami_EDKM
MSKLLKAITNLIDSNQGNSRKEGKAEMFCRSEFKKLVQQDLAPIRLSPSYRYRHIKSLPESETEPVNHKKISTVKHCVY

>Ami_EDP1_partial
MSYYGQQCKQRCLPLPICQDQLPVKCPPMCPQQWTPQYSTKSYSGYDGYCESSSLQCMEPCGPKGWLKCRPQCEQVYVPPCPP
PCVTKCPQQCVTQCVTKCPPPCVKCPPPCVKCPQPCVTQCITKCPPPCVTKCPQQCVTKCPQQCVTKCPPPCVTKCPQQXXXV
TKCPPPCVTKCPQQCVTKCPPPCVTKCPQQCVTKCPQQCVTKCPQQCVTKGQSSKVKISSNNKKYCSASKWF

>Ami_EDP2
MSSRQNQQQCKQVPTLPPALSKAIPDPVPVLDPEVPEPVPAGPEPCPAPVKEPENAPRRRQEEEHCKQPLGQPLTLAPKLEPE
PESKLGGFLVPEEPEDSVPVQLPPLVEQQQQPSL

>Ami_EDP3
MNLQKQEKQVPVSFKASEKPSPFLFSDLFSSQQQQQQWVPPKCQDKCPPKCVELCKPPKCQDKCPPKCPPQQ

>Ami_EDPE
MSSHQMQCKQKTTLPPPLCKGPPNQDQDPVPLPEPVPLPAPIPDPGQGKTPDIKIPECPPQQQQQCKLPPIIPPCPPPCKEPP
VPEPIPFPEPGPCPEKPVPLPAPMPEPGKGKTPDIKIPKCPPQQQQQCKEPPVIIPPCPPPCKEPPVPEPMPFPEPGPCPEKP
VPLPLPTPLPAPMPEPGQGKTPDIKIPECPQQQQQCKLPPIIPPCPPPCKEPPVPEPIPFPEPGPCPEKPLPLPTPIPDPGQG
KTPDIKIPECPPQQQQQCKLPPIIPPCPPPCKEPPVPEPMPFPEPEPCPEKPVPLPLPTPLPAPMPDPGQGKTPDIKIPECPP
QQQQQCKEPPVVIPPCPPPCKEPPVKCPPPCPPIQQQQQKQPCQWPPQQK

>Ami_EDPL
MSHDQQQIKQPFQPPPESSQLCPPLKFLESCPSAPPKCPEPAPPPPKGPEPSPQPPMMPTC

>Ami_EDPQ1
MSYSDQQQCKQVVCPPPVCPPTKCPPVCPPQKCPPPDCPPPVCPPQKCPPQKCPPPDCPPPKCPPQK

>Ami_EDPQ2
MSYPNQQQWQQVVQPPPVIPPQKCPPLVFPPQKCPPPQIPPPKCPVPDIPPQKCPPPQWPQQKCPPQK

>Ami_EDPQ3
MSYPNQQQCQKVVHPPPVIPPQKCPPPVIPPQKCPPPQIPPPKCPVPDFPPHKCSPPQWPQQQCPPQK

>Ami_EDPQ4
MSYPNQQQCKQVVCPPPVIPPQKCPPPQCPLPKCPPSKCPPPQWPDQKCPLPQWPDQKCPPPQCPDQKCPPPQCPQQQ

>Ami_EDPCQ
MPWNRQQCKHQPLLGSSHVNKSLWQQDCEYCPLQCPQPRGKQCMQQCLALYPLHQSVCKGSLPGMTVSPQHMCVAECLRPCES
ESLQPGCTAEQPLQQHVINSQLCCAHTAQNWHQTANSQPCCTTVTTEPQRVTKCKAPCAPSAPNQECVNNRPPCAPSAPNQKC
VNNRPPCAPSTPNQKCVNNRPPCGPPAANHSCCTKSPPCKPESPAEERQEESPLWESVPERLQLQDMTDSQGMTKCPQLLCMM
DDLPSCMHTCPALKCKTTCPPPSSAQCPALQQSLASCPPLQAIQHPPLPRATNCSLPYDATHLPPPQQCWTTCPLTHPSNSTC
PLLEQSIATCHLPPCVMEGLLPATTKYPRGRQHLHKHLPPSFMTKRFPSGMTAAARQSCLTKRRRQRILRRPLLRPRMKCPLP
QQCFAMRPLPPSASECLLPGTTECPPEQRAPAHLPQQHIAKAHPQGVTGFPKYHRK

>Ami_EDPCV
MSFQHQCKQPCLPPLICGQTVPSQPCVAPCSTVYVDPCPPGCVNPCPPQCVDPCPPGCVKPCPPQCVDPCPPVCVNPCPPQCV
DPCLPKCPPSQQCCAQTKLC

>Ami_EDQA_partial
MYYPQQHQCKQPCLPPPIVKNCPLQTVEPCGTVHVSQCATRCVDTCGTLSVAQAASGSLSPCGGISEAQAMGTSLSPGGGISE
AQAASKSVDPRGGVSVAQAGGTSLCPCGGVCVACAASRSVGPYGGVSVAQAASKSVDPHGGASVAQAASQSVDPRGGVSVAQA
GGTSLCPCGGVCVACAASRSVGPYGGVSVAQAASKSVDPRDGASTAQAAGQSVDTRGGASMAQAAGQSVDPRGGVSMTQATGT
SLCPCGGVCVACAASRSVGPYGGVSVAQAASKSVDPRGGASVAQAASQSVDPRGGVSVAQAXXXATGQSVDPYAGVSVAQAAT
KCIEPGAKGYVRPYAIQCADVCRSKCVASYGQVKVDPCAPGCAKTYPLQNVDPCLPKGSPVQQCSRKSKQC

>Ami_EDQL
MCSRENRDCHDRESSSCHDSGRSSCHGSGDVICHEVTPLPDIQPMPPMPMPTPMPAPAPIPCQQQQTKQPIHWPPQQQHQK

>Ami_EDQM1_partial
MSWQNQQGSGCYRSGGCHSRGSSGGGCHSGGGCHGGGSSGGGCHSGGGCHSRGSSSGSCHGGGSSSGGCHSXXXIGRGYYGGG
GCHGGGSIGGGCHSSGGCHSGGGYYGGRSFHVISGGHSQGSQQHKQISQVPSQKLK

>Ami_EDQM2
MSQQSQQGSGCHGSGGCHGTRSSSGGCHGGGGCHGGGGCHGGDSSGGGCHGGGSSSGGCHSGAGCHGGRFSQVIGGGHSQGSQ
QCKQISQVPSQKLK

>Ami_EDRYA
MYSPGHYTAFCHGPSGSCLGKGGPYGCGQTFCSGIQHSQGDSRSSCHSSGPLCHGSGPFDQKTWRRQHVRKRRPVWPCQAPVQ
KCCAPAQLRYA

>Ami_EDWM
MTCSSGRESYFNLNSTWYDPSGSWLENHRIPLCYADDSCCGGCNPDVRGVGGHNYRPCWYRRSVCSEAERGSSSGYCGSEDSG
CARRPTLGYSDGCGGYRRGPDRCNGECSSHEFGRRPTYHYAADVYLANERLACSEGCHGSSGGFYGSSGGCHRRRRCGEPCHG
SGSYGSSRGCHGRRRSVCGEPCHGSGSSGYLQPVCVKPEPCIPRCPPRQKYVRSTQSCCIPVQTYCAPVQVYCPPVGKYSSGR
QQCKQTSKLPTLKAK

>Ami_EDYM1
MSYYGYQLKQQCYVPPGVKYSSCVTRCPKPPATKWTTPCATKRTEPCITKKLPEPCATTCVKTRVVRCPLPCTPTCPEPCAAE
CITPCATGYLEPCGLQQPQPFSKGWEHQWAPQYIQPCPMICPPVCGPAYIQPPAQKCTAPYYFQWSNRCGYGNCGPC

>Ami_Lor_partial
MSSTQQKTACQEIPQQSRGLQGSSCHGGGSSVSSGNGIGGASSCCGGSSGQNICVSGSSGSSCCTGGSGYGIGGGFNYGGGSS
GQKIGVTGGSSSGXXXVSSGQTIVGSGSGGSDYGIGGGSSCGGVSSGHKTIVGSGSGGSGYGISGGSSYGTGGGSSSGGVSSG
HKTIVGSGSGGSSYGIGGGSSSGGVSSGHKTIVGSGSGGSGYGTGGGSSYGTGGGSSSAGVSSGHKTIVGSGSGGSGYGIGGG
SSYGIGGGSSCAGGSSGQTISIGSCSGSGGSSGISIGGPGIGGGLSSGGSGRSSSKLIINSRGSGGSCSISGSSSSGSGGSLQ
SVPQHQTKQPCQWPPQQK

>Ami_PGLYRP3
MMLRLAVLFSALCASSCQLACPPIISPAKWGSRPAKCAAPLSKVPPGNVIIIHTSGSACHTQPECSELLRNIQVFHRDMKEWC
DISYNFLIGEDGNVYEGRGWLLEGAHTYGYNDLSLGIAFIGNFTERSPNEAAWKTLKNLLTYAVQSGYLASDYLLMAHSDVSN
TISPGKLIRETIKMWPHYKH

>Ami_S100-A9
MSKKCPDAQTELEKAIETIINIFHQYSVRVGHFDTLTKMEMKLLIEKQLPNYMKNQTKPGEIDALFKDLDKNKDKQLSFGEFM
VLITRVTIATHEHLHHCGEEEGQHHHQDEHHHH

>Ami_S100-A11
MSKVPVAPTETERCIESLLAVFQRYAGRSDRDETKLSKTEFLAFMNSELASFTKNQKDPAVLDRMMKKLDLNCDGQLDFQEFL
NLIGGIAVACHDALCTGGPGGPKGSSGPKGSSGPKKL

>Ami_SCFN_partial
MPHLLDSIGTIINVFYQYATEDREGSSLSRRQMRLFIQKEFADILVNPYDPLMIDTVLRLLDQDGDGSIDFPEFLILSFRVAQ
ACYSYLAPKPGLQERQEQGRRSKELNELEAKADRGRSHQLREPEPRVGRRRHPEDPERKPEPRRDEGRRQQSLEPEQQVYEGR
RHQSHDPEQQEEVRSRSQPRDITQSHERSQRQVLEHEPPLYEESHRQPHEQECREKVRSRSQPRDTNTQAYERNRRPVLERES
QLDKESHRQPREPKKQEEVRSRFQSREPEPRWDEGRQRQPLEPKEREEVRGRSQPRDTDTQTYERSRRPVLEPEPQLYEERHH
KPREPEQREEVRGRSQPRDTNIQIYERSRHPVLEPEPQLYEERHHQPREQEQREEARSRSQPQEPEQLVYEGKGRRPHEPEER
EKVKSRSQPRDIDAESYERGQRPILEREPQLYEESHRQPREQEQQEAVRSHSQTQEPEQVYAGRRRQPLEPEQREEVRGRSQP
RDNDTQTHERSQRPVLEHEPRLYEESRRQPHEPEQREEARRRSPPQEPEQPVYERGRRQPREPEQQEEVKSRFQPCDTDTQSF
EKSRHPALEREPELYEESHRQPREPEQREKVRSHSQPQEPEPRGDEGRHRQLHELEQQEEVRGRSQPRGNDKQTHERSQHPVL
EREPQLYEESHRQPRVTEQQEEAGSCSQSREAEQQVYDGRGHQPCEPEQQEEVRSRSQPREPKPRGGERRRHLPHDPKQREEV
KSHSQPSDTDTQTYERSQHPGLECEPQLYEESHRQPREPEQQGEVRSRFQPQESEQQVYEGRRHQPRGPEQQEEVKSRSQPHN
ADTQNSERKQHPALEREPQLHEERSRQPREPEQREEVRSRFQPQEPEQQVYGRRGHQPRDPKREEVRSCTQWHETDTQNYERS
RHPVLERESQLYERSRRQPREPEQQEEVRSHFQPQEPGPRGDEGRQRQALEPEQQVYEGKRRQPLEPEQQKDIRSRFHPSDTY
TQSYVRSQRPALERESQFYEESPRRQPREPEQREKVKSRSQPQEPEPQGNERRHGQPYGPEQRAEVRSQSQPRDSDTQTYERS
QRPVLEXXXRPVLEHEPRLYEESHRQPREPEQREEVRSRSQPRNNDIQIYERSQRPVLECEPRLYEESRRQSPEPEQREEARS
RSQPQEPEQPVYDSGRHQPREPEQQEEVRSRSQPHEPEQVSRESYCEPRVPEQREVKDCYQSRESEQVYEESRHQVYEPDQQR
EVRSCYQPHVSEPRVDQGRQHQLRVSEEQYEGRRHQPHEAKTQAYERSRHPFLEREPERYEGSRIQLRELAQEGVRNRFRPRE
RGAQTDKRSHHELSEREPQVDEERCRQLCKPEHLGDVRRPYQPYEREQQVNGRRLRQSREPEQEHEGSHHQPSEAEWPAEVRT
HYLPREPEYMHEGRRRQVQDADRQGEVRSHYQPRAPDTRAFEGAHRPLFEQEPQLYERSRRQPPEPDQLGEERRHYEPRVPER
QVNEESRHQAHRIELQVYEQSRDLLCEPEPRVDEQRQRHRQEPEQQVYKRSRHQLSEVEQGEVRSHSQTREREQVPKSHHQPR
DPEQRGAKDRYLSREPEQVYEESHRQVCEPNQQGEVRRCYQPRETKPQAFKGRHCQLLECEPQLYEGRHEVKQADEGSHLPLE
PEQRETRSLYCLYDGHQHQPHQLEQRVDDGSRYQHHEPEQLRDVRSRYQPREPGPQGDARSRRPFHEREPELHEGSRRQPREQ
RDVWSHSQPREPDPRVDERSQPRPRQPELQVYEGSGHPLRDREQGESRDRSLPLDPETQTYERSRVQARDPEPQRDGQTREPR
DSELQLDEKSLCQPRAPVQLGNGRRRYQPRELEPEANEGSCYQPRGPEQQRNLQSRGQPREPEQRVKEGSRSHLPEQVQQGDA

QSRYVPLETRPQAHDGSRRQLQEPEIQHYEGNCHQSQNPGQQGDVRSHYRPHEPAQQVHKGNRQELHESETQKGEGSQRQPRE
PKEQVYEGSHRPTPLCDPEPQRAVQNSGQSRDPTPQQEEVNCHQPREPEPRGVAGNVHQRHEVLPPRDDQSLLPPDQPVPQRG
DSSDDPQPEVRPHDRSLPQPLEPERQGNERTQRQTLEPERREAEKNGQRPYAPDPKRDDEICHLVSAKQRDDGGRQPQSRETE
PRGGTGTRLQQRESEAQGNTATSRNPREPEAQGPERARQPRYPESQGVESSRQPQKAAPQEGERDNQLPQKAEPQDGERNRPQ
AGEAKPSKEEASQREPHNPNSMDDNRSRAAPTPPPTGDPGSQTQPREGEPRDGSRHQSNRPEGRGDEGSQPRAYEPGCRAGAT
VPSQTPERGLREGEGSRQQPWALEAPAEGGSRQQPPQGDAASQRQQEIAPEQAEGSHHLPREPLAQLQEESPTRAAESQEGEQ
SHHPPEPVVSQERLGDPSLDEAKASLPCSPLYVYLLAQKAEQQLCPVPAPQEQP

B

>Ami_EDbeta1
MTCSPALSSGICASPCGVAVPQPITDSWNEPCVRQCPDSTVVIQPPPSVVTIPGAILSSFPQEGIVGSTGAPHVAAGFGGGFG
SQGFYGSRAYMGAGGPYGYGGIWGYGGLCGSGGWRSGHRYLNGNCAPC

>Ami_EDbeta2
MACSTNVCNNSSVSCGVAAPQPIADSCNEPCVRQCPDSTVVIYPPPVVLTFPGPILSCFPQESVVGSSASPVLGSSLGGSYGA
GYPYGGSRCGSRYSNGSCGPC

>Ami_Beta1
MSCGTGCSNPCEVNCPQPQAVTANEPCVITCPDSRVIIYPPPVVVTFPGPILTTCPQESVVASTASADTVPAEMPASVPLTAA
VSGSLEPRAETIAPPIIPRPLPRYVPKYSHTYSSHWMHPCNTNRFGKRWAY

>Ami_Beta2
MSCYDISYPPCGVTLPPCPEPFAVTSNEIYSAQYPDRIVETELEDGQPCTVTYPGPILTTFPQQTLVGSSALFDMERLLGSRR
SFEFEGLLGLGGICGPGSLCNSEPFDDFPYGNCGPV

>Ami_Beta3
MACTDLCYPSSGIACPTPIANSYNDLCVRQCPDSRAVIQPPPVVVTFPGPILSSFPQDSIVGSSGAPVVGGYGSSFGTRFGYS
GLEGSLGYGSSGGYGGSGYEGLGGGYVGGSLGYGSGSLGYGGGLCSSGSLYNYGRLYGSGFGYGYCSPFSYRRYNRYRRGSCG
PC

>Ami_Beta4
MSCYDECYVPCRATCPSPVADSCNELPCVRQCPDSTTVIQPPPVVVTFPGPILSSCPQGSVVGLLGAPSTGSSAGSLSYVGSF
GSGGLYNYGGLYSSGLSGLGTGDCCPYSRPLNTYHYGRCFPC

>Ami_Beta5
MSCTDLCYPSGGIACPKPYADSCNEARVRQCPDSRVVIWPPPAVVTFPGPILSNFPQDSIVGSTGVPAVGHGAAGGTALSNGI
GGAGGFYGYEASLDSGGLYGYGGSLGYGGLCGYGGLSSDSGSCYSSGYCSPYSYR

>Ami_Beta6
MSCTDLCYPSSGIACPRPYADSCNEACVRQCPDSRAVIQPPPVVVTFPGPILSNFPQDSIVGSVGVPTVGHGAAGGTALSNGT
GGVGGIYGYGAYLGSGGLYDYGGSLVYGGGYGGSLGSGGLCGYRGSLGYGGPCGYGGLSSGSGSCYSSGYCSPYSYRRYGRYR
YGSCGPC

>Ami_Beta7
MSSYGQLISSRCYNPCEVTCPRPYADAWNEPCVTSCGDSRAVVYPPPVAITFPGPILTSCPQDSYVGTSEPQCIGSPYAAGGY
LGYRGSVGTGYSYPSYSRQLNRYHYGGCGPC

>Ami_Beta8
MSSYGQLISSRCYNPCEVTCPRPYADAWNEPCVTSCGDSRAVVYPPPVAITFPGPILASCPQESYVGTSEPQCIGGPYTAGGY
LGYRGSVGTGYSYPSYSRQLNRYRYGGCGPC

>Ami_Beta9
MSSYGQLISSRCYNPCEVTCPRPYADAWNEPCVTSCGDSRAMVYPPPVAITFPGPILASCPQESYVGTSEPQCIGGP
YTAGGYLGYRGSVGTGYSYPSYSRQLNRYSYGGCGPC

>Ami_Beta10
MSSYRQLISSRCYNPCEVTCPQPYADAWNEPCVTSCGDSRAVVYPPPVAITFPGPILASCPQESYVGTSEPQCIGGPYTFGGY
LGYRGSVGTGYSYPSYSCQINRYRYGGCRPC

>Ami_Beta11
MSAYGQLISSRCYNPCEVTCPRPYADAWNEPCVTSCGDSRAVIYPPLVAITFPGPILASCPQESYVGTSEPQCVRVPYTAGSY
LGYRGSASTGYSYPSYSRQLNRYQYGGCGPC

>Ami_Beta12
MSSYGQLISSRCYNPCEVTCPRPYADAWNEPCVTSCGDSRAVVYPPPAAITFPGPILASCPQESYVGTSEPQCIGLPYTADGY
LGYRGSVGTGYSYPSYSRQLNRYRYGGCGPF

>Ami_Beta13
MSLYRQLISSRCSNPCEVTCPQPYADAWNQPCVTSCGDSRAVVYPPPVVITFPGPILSSCPQESYVGSSAPISIGSSFGYGGS
FTYGGSLGYGGSCSTGSTYPCYSQRVNRYRYRSCGPCQTQKEFTCTRNIQETKCKIQAQGLADDCEKC

>Ami_Beta14
MSFNRQLLSSRCFNPCEVTCPQPYANAWNEPCVTSCGDSRAVVYPPPVVITFPGPILSSCPQESYVGTSEPLQIGSSFVSRGS
VGSGSSLGCLSPYYSQRYNKYRYDNCGSC

>Ami_Beta15_partial
MTLTGALCCYQPRPPCDVVCPRPYADAWNEPCVKSCGDSRAVVHPPPVVVTFPGPILASCPQESYVGTSLPEVNGGSFGSGSS
FGAGFGYRGNLGSRGYLGYGGGSLGYGGGSLGYGGGSLGYGGXXX

>Ami_Beta16
MSCSKNPCNDSCSTRCEAKCPEPQVITSNEPCVIACEDTRVIIYPPPVVVTFPGPILTTCPQETVVASTVTLAESSDDATLAE
SPAMIPSAPEVTRSSVPCHEICPPCIIPRPKPQYLPNYSYTFSTQWIHPCNRSGFKKYKSS

>Ami_Beta17
MSQSLSSRCLPPCDMTCPRPCADAWNWPCVTSCGDSRAVVYPPPVVVNFPGPILASCPQESIVGTVLPRPSGDIGPFPYGYGG
GYGSVSSYGFGGGYRSGSDYGSGSCYGSVSSRRYRKFSSGSCGPC

>Ami_Beta18
MSQSLSSRCLPPCDVTCPKPCVNAWNWPCVTSCGDSRAVVYPPPVVVNFPGPILASCPQDSIVGTVLPRSSGGIGPFPYGSGG
GYGSGSSYGSGGGYGIGSGSGYGGSYGMGSGYGSGGSYGVSSGYGSGYGSGFDCGFGSGYGYGGGYRSGSSYGSVSSRRCRRY
SSASCGPC

>Ami_Beta19
MALSSRCCPSDICPKPCVDACNWPCVTSCGDSKAVVYAPPVIVHFPGPILTSCPQESIVGTSMPNIIRGGGGPYSSGTIGSGF
SSGFSSGFSSGSGSGFGGGYGLGGGYGLGGGYGIGGGYGMGGGYGIGGCYGFGGGYKSGGYYGSVSSRRRRFSSVGCGPC

>Ami_Beta20
MSCGQISSRCLPPCEMMCPEPYVAACNYPNTTSFGDSRAVVFAPPVIMTFPGPILASCPQESVVGAAEPYPIGGYPGGPYEGS
GGSYGGSSGIGGSYGASYGHSGGSYGYSGGSYGGSSGIGGSYGTSGRSYGGSSRTGGSSGATYGSSGHSSGISGGSRSSGTSY
GGSGGSRGGSRGGSGRTGVSSVSSGDPYGISGGSHRSGSSSGGSGESPKRYGESHERPGESEHSSGSFGGSYGISGGSRHSGT
SSGGSGESPKGYGESHEGPCESEHSSGRSGGSYGICGGSRRSGTASGGFGESHEGSRRNEDSSESSKGFYGISGESYGSGDPY
EGYGGSYAVGGSGIFRSSFFSRCPPGSFSSTFPFTRFAYQRQFGNNEFF

>Ami_Beta21
MSCNTDPCPEGRPSPCEVKCPQPIVTSTNEACVVSCGDSRVIIYPPPVIVTFPGPILSTCPQESLVGAAVPCESGVSQSATTV
PLTSEIGGNSGFSVPLRSEIGGNSGFSVPLRSEIMGNSGCSAERLYLNREPEQPSTYTYSFTSQWRHPCNRPGWNRYRSFYMK
KEEPEEEEKPKEWHVGTEESS

Supplementary Figure S1. Amino acid sequences of proteins encoded by EDC genes of the American alligator (Ami, *A. mississippiensis*). **(A)** Amino acid sequences of EDC proteins apart from corneous beta proteins (CBPs). **(B)** Amino acid sequences of CBPs, also called beta-keratins. Several amino acid residues are highlighted to indicate the peculiar amino acid compositions of SEDC and SFTP proteins (Fig. 5). Cysteine (C) is highlighted in yellow, proline (P) in green, lysine (K) in cyan and glutamine (Q) in grey. Serine (S) and glycine (G) are shown with bold fonts colored red and orange, respectively. Three X´s indicate an unknown numbers of amino acid residues due to gaps in the corresponding gene sequences. Among S100A proteins, only those encoded by genes flanking *PGLYRP3* and *Scfn* are are shown here.

A

>Cpo_CRNN
MTQLQGNIEGIISAFNAYAKEDGGCITLSKGELRQLIQQEFPDVLVKPHDLQTIDQVLQHLDAESEDRIDFDEFLVLVFQVAKACHKEL
NPCQPSGDGQSSASQGDASRGQAQRAEEDPGRRHIQEPQALEQGQKQPEAPEQDASRPQAPETPTAEGDLSHRHTQDPEVSQGDGNREA
QAAETPEHDSVRRQGQELEQDPSHHRAQEQDPNREGQDPQVPQQDVTHEALESGALEQAPNRHPALQSSTSETDLDRHQGLQSEAPEQD
LSPTAERESTHYEAHEPQAPEQGSQESQPAEQDQNRHQSEEPETLEHTLRHQPQEAQPTKQDLTQETQTPRVPEGDVSRGGTPFSPALQ
QDRGAQQDPREEALTAYRPYIYQCQTPPTFPYQWLPKQ

>Cpo_EDAA1
MSDSLGMLEDLCYQNSSCCWRPHCRRPCYCCCYDPCTGELIWEGWCCCPGWRGSGRYGSCWPC

>Cpo_EDAA2
MSDSLGMLEDLYYQDPSCCWRPRRRRPCYCCCYDPCTGELLWEGWCCCPCWPWRGKYGRRWPC

>Cpo_EDAA3
MSDSLGMLEDLCYQDSSCCWRPRRRRPCYCCCYDPCTGELIWEGWCCCLGWRGSGSYGSCWPC

>Cpo_EDAA4
MLEDLCYQGLDCGWRSRYGRPCYCCCYDPCTEELIWEGWCSCPWWRGRGRYGRCWPC

>Cpo_EDAA5
MFDSLDTLDDLFYQGQSDCWPPTPKRPPYTCCCYDRWGRLVWRGCCWSIPPWWCRTKIPGKGWPC

>Cpo_EDAA6
MFDSLDTIEDLYYPGQLDCTPRYPRRRYWCCSYDRCGRLVWQGPCCYPGPWCPHKGSSGSSWPC

>CPo_EDAA7
MFDSLDMIKDLSYQGQSDCFSHQHRRPPYTCCCYDQCGRLVWRGCCWSIPPWWCCQGSSGSSWPY

>Cpo_EDAA8
MFDSLDAIEDLCYQGQYDCWDPCYRRPYWYGCWDPCTYRRPYNYGNCYGYGGLYRLGGCYPYSSRWGRKYSYGNCWPC

>Cpo_EDAA9
MSESLDMLENLWYPGQSNCWDPWYRRPYWNSCWDPCTYRRPYIYDNCYGYGGLYGFGRCYPYSTRWGRRGSWGSCWPC

>Cpo_EDAA10
MFDSLDMIEDLPYQGQSDYFPHYCRRPTYMCCCYDWSGVVAAAPYNPGWCCRSSSGNCWLC

>Cpo_EDAA11
MSDSLDMLENLWYPGQSNCWDPWYRRPYWNSCWDPCTYRRPSWSGCWDPCTYRRPYIYNSCYGYGGLYGAGGCYPYTTRWGRRYSAGSC
WPC

>Cpo_EDAA12
MTYHQSGSDDMCYTPCSYGGLYGCQSLTGCWKPWTYRRPHSYGCWNPCTYRWPDSYWDPRGYGSG

>Cpo_EDAA13_partial
MSESLDMLENLWYPGQSNCWDPWYRRPYWNSCWDPCTYRRPYWSGCWDPCTYRRPYIYXXX

>Cpo_EDAA14_partial
XXXGCWDPWYRRSSWSGCWDPWYRRSSWRGCWDPCGYGRPYIYGLGGLSGSGSCYPYYSRWGRRGSYGKCWPC

>Cpo_EDAA15_partial
MFDSLDTTEDLYYKGQSGCWDPWYRRSSWSGCWDPWYRRSSWXXX

>Cpo_EDCH1
MCSRGSCHDHGSSSHGCHGRESSCHGSSSSINCVIEKPVPVCPVPQCCPQLPQCCVPVQPCCPPVQCCQQSKQCFKCPPQCPK

>Cpo_EDCH2
MCSRGSCHDHRSSSHGCHSHESSCHGSTSSINCVIEKPVPVCPVPQCCPQLPQCCVPTQQCCPPVQQCCPPVQCCQQSKQCCKIPPPCP
K

>Cpo_EDCH3
MCSRRSCHDHGSSSHGCHGHESSCHSSSTSVNCVIEKPVPICPVPQCCPQLPQCCVPTQQCCPPVQCCQQSKQCCKIPPPCPK

>Cpo_EDCH4
MCSRRSCHDHGSSSHGCHGHESSCHSSSTSINCVIEKPVPVCPVPQCCPQLPQCCVPTQQCCPPVQCCQQSKQCCKIPPPCPK

>Cpo_EDCH5
MCSRRSCHDHGSSSHGCHRHESSCHGSSSSLHCVIEKPVPICPVQPCCPPVQQCCPPVQKCCPPVQQCCPPMKCCQQSKQCCKIPPPCP
K

>Cpo_EDCH6_partial
XXXSPVKCCQQTKQCCKFPPPCPK

>Cpo_EDCH7
MCSRRSCHDHGSSSHGCHGHESSCHGSSSSLHCVIEKPVPICPVQPCCPPVQQCCPPMQKCCPPVQKCCPPVKCCQQSKQCCKIPPPCP
K

>Cpo_EDCH8_partial
XXXPVQKCCPPVKCCQQSKQCCKIPPPCPK

>Cpo_EDCH9_partial
MCSRGSCHDHGSSSHGCHGHESSCHGSSSSLHCVIEKPVPICPVQPCCPPVQQCCPPVPKCCLWQASRGRXXX

>Cpo_EDCH10
MCSRGSCHDHGSSSHGCHGHESSCHSSSSSVNCIIEKPVPICPMPQCCPPVPQCCPPVQQCCPPVQQCCPPVQCCQQSQQCCKIPPQCP
K

>Cpo_EDCH11
MCSRGSCHSHHNSCHGSSSHCHEPRRCCNVVVVEKPYVQACCPVPCYCPPVSCCYPRYQSSQCCKFPQYPKCPPQYPK

>Cpo_EDCH12
MCSHGSCHSHRNSCHGSSSRCHEPRCSCNIVVVEKPYVQACCPVPRYCPVPSYCPPVSCCYPRYQSSQCCKFPQYPK

>Cpo_EDCH13_partial
XXXHEPTCSCNVVVVEKPYVQACCPVPCYCPPVSCCYPRYQSSQCCKFPQYPKCSPQYPK

>Cpo_EDCH14
MCSHGSCHSHHNSCHGSSSRCHEPRRSCNIVVVEKPYVQACCPVPRYCPVPSYCPPVSCCYPRYQSSQCCKFPQYPK

>Cpo_EDCH15
MCSRGSCHDHHRSCHGSQYGSHCQEFRPSCNITVVERPSVQAWCPVQQFCPVQCYRSPTCCYPRYQYSQCCKFPPQYPKCPAQYPI

>Cpo_EDCH16
MCSRRSCHDRDTSCHRSRPSCHSSGSGCHESRRSCHIPVVERPPYCQWQQYRPVQPYYPGYQYSQCCKFPQYPQYPQYPK

>Cpo_EDCH17
MCSRRPCHERETSCHESRPSCHSSGSRCHESRPSCHISVVERPSYCQWQQYRPPYYPDYQQCYKYPVQYPKFPQCPQYPQYPKYPQFPQ
YPK

>Cpo_EDCRPL1
MCSCCSGCHGTGSVQPICYVQPVCCELVYIQRSSGSCCQPCGSCCGSCCGGSRSCPRVVMQRCPMLVCCPPLQYLAPMQQCWLPLKKC

>Cpo_EDDML
MAFPNQQQCKQPCLPSLVCIQKCPPGCVDQRDAACVKKHTDPCGNICTKSCTTKCVDSCNDISTMLCVTKCVDPCGAACVKECTTKCMC
PSNTVCEKPCVTKYVDPCGTSCVTSCVTPCPEPGNTVCVKECITKCMDPCGTFCAEPYVTKCVDPSCSSSAKLCITKCVDLCNTVCVKE
CTTKCVDPCSTRCAKPCVTNCVDLCGTVCAKPCITKCVDSCCTGCGKLCVTKYMDPCGTICAKPCLTMCMNPCSTRCAKPTVTKCVELC
STVCPKPCIAKHGDPCGTICVKECTTKCMDPHDVICTKPCVTKCVDPCTTSCVTSCVTKCTESCNTVCIKKCTVKCMDTCSTVCAKPFV
PKCMDPCCPRCTASSGTMCMDPCAPVCKKTYPLQSVDPHLPKRPPVQQCCQKPKQC

>Cpo_EDKM
MSKLLKAITDLIDSNQGNSRKEGKAEMFCRSEFKKLIQQDLAPVTLSSSYRYRHIKSLAESETEPVNHKEISTAKHCAY

>Cpo_EDP1_partial
MSYYGQQCKQRCLPTPICQDQLPVKCPPMCPQQWTPQYSTKSYSGYDGYCESSSLQCIEPCGPKGWQKCRPQCEQVCLPPCPLPCITKC
PPPCVKCPQQCVTKCPPPXXXQCVTECVTKCPQQCVTKCPQQCVTECVTKCPPPCVTKCPPPCVTKCPQQCVTECVTKCPPPCVTKCPQ
QCVTECVTKCPQQCVTKCPQQCVTKGQSSKVKISSNNKKYCSASQWFW

>Cpo_EDP2
MSSRQNQQQCKQVPTLPPALSKVIPDPAPVLDPAVPEPVPAELEPCAAPVEDLENAPGRHQEEEYCKQPLGQPLILAPKLEPEPESKLG
GFLIPEEPEASAPVQPSPLVEQQQQQQQPPPSL

>Cpo_EDP3
MSSQQQQQQQERVPPKCQDKCPPKCVELCKPPKCQDKCPPKCPSQQQQQQQQQRPKQK

>Cpo_EDPCQ
MPEDALYRQQCKHQPLTGSSHVNKSLRQQVSEYCPLQCPRLRVKKRVALYPLHQSVCGALLPSMTVSSQHMCVAECLWPCETECLQPGC

MAEQPLQQHVTNSQLRCMHTAQNWHQTVNSQPCCTSVTTEPRRATKCKAPCAPSSPNQECENNRPPCAPSAPNQKCVNNRPPCAPSTPN
QKCINNCPPCGPPAANHSCCAKRPPGMPESPAEKGQEESPLWESGPERLQLQDMTENQGMTKCPQLLCMTHDLPPCMHTCPALQCRTTC
PPPSSAKCPALQQCLASCPLLQAIQHPLLPKATSCPLPYDATHLPPPQQCWTTCPLLNSSNSTCPLLEQRITTCHWPPCVTKGMLPATT
KYPRQRQHLRKHLPPSFVTKRLPSGMTAAALQRGVTKWHRQCILKRPSLQPRMKRPLPQQCFAMHPVLPCVSEYLLPGTTECPPEQRAP
AHLPQQRITKAHPPGVTGFPQYHRKRGFEGILGGHI

>Cpo_EDPE
MSSHQMQCKQKTTLPPPLCKEPPNPGQDVVPLPDPVPLPAPMPEPGPGKTPDIKIPECPLPQQQQCKQAPIIPPCPPPCKEPQVPEPVP
FPEPGPCPEKPVPLPAPVPEPGKGKTPDIKIPECPPQQQQQCKQPPVIIPPCPPPCKEPPVQEPMPFPEPGPCPENPVPLPAPVPDPGQ
GKTPDIKIPECPPQQQEQCKQPPVIIPPCPPPCKETPVPVPCPEKPVPLPVPVPDLGQGKTPDIKIPECPPQQQQQCKQPPVIIPPCPP
PCKEPQVPEPMPFPEPGPCPENPVPLPAPVPDPGQGKTPDIKIPECPPQQQEQCKQLPVIPPCPPPCKEPPVKCPPPQQTCPPIQQQQQ
KQPCQWPPQQK

>Cpo_EDPL
MSHDQQQIKQPLQPPPESSKLCPHLKFLESCPSAPPKCPEPAPPPPKGLEPSPQPLLCPPAEKGHMAEPF

>Cpo_EDPQ1
MSYPNQQQCKQVVCPPPVCPPTKCPPQKCPPPDCPPLKCPPQKCPPPVCPPQKCPPPECPQKCPPQK

>Cpo_EDPQ2
MSYPNQQQCKQVVCPPPLCPPPKCSPPVCPPPKCTPPDWPDQKCPPPKCPPPKCPPPYWPDQKCPPQK

>Cpo_EDQA_partial
MSYPHQHQCKQPCLPPPIVKNCPLQTVEPCGTVHVSQCTTRCVDTCSTASIAQAASRSLSPCGGISEAQAMGTSLSPCGGVSVAQAASR
SVDPCGGVCVAQAASKSVDPCGGVSVAQAVGTSLCPCGGVCVACAASRNVRPCGGVSVAQAASKSVDPCGGVSVAQAAGTXXXCGGVSV
AQAASRSVDPCGGVCVAQAASKSVDLCGGASMAQAKGTSLSPCAGASVAQATTKCIEPGAKGCVRPYAIQCADVCRPKCVASYGQVKVD
PCASGCAKTYPLQNVDPCLPKGSPVLQSKKC

>Cpo_EDQL
MCSRENRGCHDTESSSCHDSGRSSCHSSGDVICHEVTPLPDIQTVPPMPLPLPAPAPIPCQQQQTKQPIHWPPQQQHQK

>Cpo_EDQM1
MSQQNQQGSGCHRSGGCHGRGSSGGSYYSGGGCHGGGSSGGSCYGGGGCRGGGSSGGRCYGGGSSGGSCHGGGSYHGGGGSGGRCYGGG
GCYSGGSIGRGCHGGGSSGGGCHSGGGCHSGGGCHSGRSFHVISGGHFQGSRQHKQISHVPSQKLK

>Cpo_EDQM2
MSQQNQQGSGCHGGGGCHSRRSSSGGCHGGGDCHSGGSSGGSCHGGGSSGGGCHGGGGCHSGGGCHGGGSSGGGCHGGGGCHGGGSSGG
GCHGGGGCHGGRSSQVIIRRHSQGSQQCKQISQVLSQKLK

>Cpo_EDRYA
MYSPGHYAAFCHGSSGSCLGKGGPYGCGQAFCSGIQHSQGDASSSCHSSGPLCHGSGPFDHKAWRRQHVRKRRPVWLCQAPVQKCCTPA
QLRYA

>Cpo_EDWM
MICSSGRESYFNLNSTWYDPSGSWLENHRIPLRYADDSCCGGCNPDVRGVGGHNYRPYWYRRSVCSEAERGSSSGYCGSEDSGCARRPT
LGYSEGCDCYRRRPDCCNGECSSHEFGRRPTYHYAADVYLANERLACSDGCHGSSGGFYGSSGGCHRRRRCGEPCHGSGSYGFSRGCHG
RCRSVCGEPCHDSGSSSHLLPVCVKPEPCIPRCPPKQKYVRSTQSCCIPVQSYCTPVKACCPPIQAYCPPVGKYSSGGQQCKQTSKLPT
LKAK

>Cpo_EDYM1
MSYYGYQLKQQCYVPPGVKYSSCVTRCPKPPAMKWTTPCTTKCTEPCIAKKLPEPCATTCVKTRVVRCPLPCTPTCPEPCAAKCVTPCA
TGYLEPCGLQQPQPFSEGWEHQWAPQYIWPCPTRCPSACGPAYMQPPAQKCTAPYYFQWSNRHGYGNCGPC

>Cpo_LOR_partial
MSSSQQKTACQEIPHQSGGLQGSTCHGGGSSVSSGCGIGGGSSYGGGSSGQKIGVTGGSSSGSSYGGGLSSGISGGSAQKVVIAGGSSG
GCSCCSSGGSSYGIGGGSSCGSVTSGQTTVVGSGSGGSSYGSGGGSSCSGVSSGQKTIVGSGSGGSSYGSGGGSSCGGVSSGQTTVVGS
GSGGSSYGIGGGSSCGGVSXXXSYGISGGSSYGSVSSGQTTVVGSGSGGSSYGSGGGSSCGGVSSGQTIVVGSGSGGSSCGVSGGSSYG
TGGGSNCGGVTSGQKTIVDSGSGGSSYGIGGGSSCGGVSSGQTTVVGSGSGGSGYGIGGGSSHGSGSSGQKTIVGSGSRGSSYGISGGS
SYGRGGGSNCGGVSSGHKTIVGSGSAGSGYGIGGGSSYGIGGGSSCGGGSSGQTVSIGSGGSSCGSGGSSGISIGGPGIGGGLSSGGSG
GSLSKVITTSGGSGGSSGGSLQSVPQHQTKQPCQWPPQQK

>Cpo_PGLYRP3
MMLRLAVLFSALCAASCQLACPPIVSPAKWGSRPAKCASPLSKVPPGNVVIIHTAGSACHTQPECSELLRNIQVFHRDMKEWCDISYNF
LIGEDGNVYEGRGWLLEGAHTYGYNDLSLGIAFIGNFTERSPNEAAWKALKNLLAYAVQSGYLASDYLLMAHSDVSNTISPGKLIRETI
KLWPHYKH

>Cpo_S100A9_partial
XXXKCPEAQTELEQSIEKIIDVFHVYSVRVGHFDTLTKMELKLLIQKQLPNYLKNQTSPGQIDALFKDLDKNKDQQLSFGEFMVLITRV
TIATHEHLHHCGEGEGQHQHQDEHHHH

>Cpo_S100A11_partial
XXXSKIPVAPTETERCIESLLAVFQRYAGRSDRDETMLSKTEFLAFMNSELASFTKNQKDPAVLDRMMKKLDLNCDGQLDFQEFLNLIG
GIAVACHEALCKGGPGGPKGPGGPKKL

>Cpo_SCFN
MPRVLDSIGTIIKVFYQYATEDREGSRLSRRQMRLFIQKEFADVLVKPYDPLTIDMVLRLLDQDGDGSIDFSEFLILAFRVAQACYSYL
APKPELQERQQQGRRGKELNEPEAKADRGRGHQLREPEPRVGRRSHSEDPEQEPEPRRDEGRQRQSLEPEQQVYEGRRHQSRDPEWREE
VRSRSQPRDIMQSHERSQRQLLEHEPQLYEESHHRPHERECREEVRSHSQPRDTSTQAYERSRRPLLERESQLDKESHHQPREPEKQEE
AWSRFQSRKPEPQWDEGRQRQPLEPKERQEARGRSQLHDTDTQSYERSQRPVLEPEPQLYEENRHQPHEREQRQEAKSRSQLQEPEQPV
YEGRGQQPHEPEEGKKVKSWSQPCDNDAQNYERGQYSICEREPQLYEESRRQPCNQEQQEAVRSHSQTQESEQVYAGRRHQPLEPEQRE
QVRGRSQPRDTDTKSYERSRCPALKPEPQLYEESRHQPREQEQREEVRSHFQPRQPEPQRDESRQRQPLEPEQQVYEGRRHQPQEPKQR
EEVRGRSQPRNAETQSHERSQCPVFEREPRLYEESRRQPREPEQQEEVRNCSQPCDNDTQTYERSQRPVLECEPRLYKESRRQPREPEQ
REEARTRSQPQEPEQPVYKGGRCQPREPEQREKVRSCSQPQDPEPRGNERRHRQLHEPEQRAEMRSHSQPCGSDKQTYERSQHPVLERE
PQPYKESRRQPRETEQQKEAGSHSQPQEAEQQVYDGRGHQPREPQQREEVRSHSQPRGTNTPTYERSQRPVLEHESQLYKGSHCQPLET
EQREEVRSRFQPREPKLRGEEGRRHLPHDPEQREEVKSHSQPSDIDTQTYERSQHPVLECEPQLYEESHHQPREPEQRIEVRSRFQPQE
SKRQVYEGRYHQPHGPEQQEEVRSRSQPHDADTQNIERNQRPALELEPQLHEKRSHQPREPEQRVEVRSRFQPQAPEQQVYGRRGHQPR
DPKRDEVRSCTQWHETDTQNYERSRHPVLEHESQLYERSHQQPREPEQQEEVRSHFQPQESEPRGNEGRRLALEPEQQVYEGRRHQPLE
SELQKDIRSRFHPSNTYTQSYVRSQHPALERESQFHDESPLQPREPEQREKVRSRSQPQEPEPQGDEGRHRQPHRLEQRAQVRSHSQPH
DTYRQTYEKNRHPILERESQLYERSRHQPLETHHREELRSRFQPQEPEPRGDEGRQRQSLEPEQQVYEERHHQPREPEQREEVRSRSQP
RNAETQSHERSQCPVFEREPRLYEESHRQPREPEQREEVRSRSQPRDNDTQTYERSQRPVLERESRLYKESHHQRRDPEQREEARTRSQ
PQEPEQPVYDGGRHQPREPEQQEEVRSHSQPHKPEHVSGESYCQPRDPEQREVKDCYQSREPEQVYEGSRHQVFEPDQQREVRSCYQPH
ISKPRVNQGGQHQLRVSEERYEGRRHQPHEAETQAYERSRHPFLECEPEEYEGSHIQLRELAQEGVRNRYWPRERGAQTDERRHHELRE
REPQVDEERCRQLCEPEHLGDVRRPYQPYEREQVDGRRLRQSREPEQEHQPSEAEWPEEVRTRYLPREPEYVHEGRRHQVQDTDRQGEV
RSRYQPREPDTRAFERAPRPLFEQKPQLYERSQRQPPEPDQLEKERCHYKPHVPERQVNAESRHQAHRTELQAYERSRDLLHEPESRVD
EQRLRRHQDPEQQVYERSCHQLSEVEQGEVRSHSQPHEHQQVPKKSLHQPWDPEQRGAKDRYQSREPEQVYEGSHHQVCQPNQQGEVRS
CYQPHETEPQAFKGRHYQLLECEPKLHEGRHEVKQVDEGNHLPLEPQQRETRSLYCLYNGQRQPHQLEQRVDDGSRYQHHEPEQLRDGR
SHYQPREPGPRGDARSRRPFHECEPEVHEGSHHQPREQRDVWSHSQPREPDPRVDERSQPRPRQPELQVYEGSRHQLRDREQGELRDRS
LPLDPETQTYERSRAQAHDPEPQRDGQTREPCDTELQLDEKSLGQPRASIQLGNGRRRYQPRELEPEANEGSCYQPRGPEQQRNSQSHG
QPHEPEQRVKEGSRSHLPEQVQQGDAQPHYVPLETRPQAHDGSRRTLQEPKIQHYEGTRHQSQNPEQKGDVRSHYQPREPTQQVHKGNR
RELRESETQKGEGSQRQPREPKEQVYEGSHRPTPLCDPEQQRAVQSSGQSRDPTPQQDEVNCHQPHEPEPRVVAGNVRQRYEVLPPRDD
QSLLQPDQLVPQRGDRSDDPQPEVKPHDRSLPQSLEPERQSNERTQHQTLEPEHREAEKNGQRPYTPDPKRDGEICHLVSAKQRDDGGR
QPQSHETEPRGGTGTCLQQREAEAQGSTTTSRNPQEPEAQGPERARQPRYPESQGVKSSPHQPQKAAPQEAERDNQPPQNTEPQDGERS
RPQAGEAKPSKEEASQSEPHNPDSTDDNRSRAAPAPPPTGDPGSQTQPREGELRDGSRHQSSRPEGRGDEGSQPQAYEPGCRAVPSQTP
ERGLREGEGSRQQPQALEAPAEGGSRQQPPQGDAASRRQQEVAPEEAAGSHLPREALAQLQEESPTRAAESQEGEQSHHPPEPVVSQEG
LGDPRLDEAKASLPCSPLYVYLLAQKAEQQLCSAPAPQEQP

B

>Cpo_Edbeta1
MACSTNVCNNSAVSCGVAAPQPIADSCNEPCVRQCPDSKVLIYPPPVVVTFPGPIITSFPQESVVGSSAAPVCGSSLGGSYGPGHPYGG
SQWGSRYINGSCGPC

>Cpo_EDbeta2
MACSPALSSGICASPCGVAVPQPIADSYNEPCVRQCPDSRVVIQPPASVVTFPGATLSSFPQDSFVGSAGLPHLGAGFGGSFGSRGFYG
SPGYTGAGGPYGYGGLWGYGGLCGSGGLRWGHRYLNGNCGPC

>Cpo_Beta1
MACPPALSSGICASPCGVAVPQPIADSYNEPCVRQCPDSRVVIQPPASVVTFPGATLSSFPQDSFVGSAGLPHLGAGYGGSFGSRGFYG
SPGYFGAGGPYGYGGLWGYGGLCGSGGWRWGHRYLNGNCAPC

>Cpo_Beta2
MSSVTGCSNPCEVSCPQPQAVTANEPCVITCPDSRVIIYPPPVVVTFPGPILTTCPQESVVASADTVPAELPAAAPLTTEVSGSLEPCA
ETVAPPVIPRRLPRYVPKYSYTYSSQWMHPCNTNRFGKRWAY

>Cpo_Beta3
MSCYDIPYPPCGVTLPPCPEPFAVTSSEIHAVQYPDRIVETELEDGQPCTVIYPGPILTTFPQQTLVGSSALFDMERLLGSRRSFEFEG
PLGLGGICGPGSLCNSEPFGDFPYGNCGPV

>Cpo_Beta4
MACTDLCYPSSDIVCPTPIANSYNDLCVRQCPDSRAVIQPPPVVVTFPGPILNSFPQESIVGSSGAPVVGDYGSSFGARFGYSGLGGSL
GYGSYGGYGGDGYEGLGGGYVGGSLGYGSGSLGYGSGLCGSGSLYNYGRLYGSGFGYGYCSPYSYRRYNRYRRGSCGPC

>Cpo_Beta5
MFCSNECYTPCTVPCPQPTANSCNEPCVSQCPDSTVVIYPPPIVVSFPGPILSSCPQGSIVGFSGPARIGGSGSSGSSLAIRVGYENSG
LSSSRWINRYHLGSCGPC

>Cpo_Beta6
MSCYNECYTPGAVACPRPIADSWNGLCVRQCPASRVVIQPPPAVVTVPGPILSNYPQDSVVGSAGVPAVGYSPRGYLGYGGSEGALVSG

GSGGALVSGGALGYGSDVGYGVGLGYRGGLGYGLGLGYGGSLGYSGGLCYGDGLGYGSYGGCYGSRSLSSYGGLCGSGYSGFGSGYCRP
FSYRRYNRSLSGSCGPC

>Cpo_Beta7
MSCTDLCYPSSGIACPRPFADSCNEACIRQCPDSRAVIQPPPVVVTLPGPILSSFPQDSIVGSAGVPAVGHGAAGGTALSGGPSGPGGH
LGYGGLYGSLGGYGGLGGYGGLGGYGGLGGYGGLGGYGGLGGYGGWGGSCYGLGSGYGYGRCYVPTYCGPYYSRRSYYGICRPC

>Cpo_Beta8_partial
MSCTDLCYPSSGIACPRPFADSCNEACIRQCPDSRAVIQPPPVVVTIPGPILSNFPQXXX

>Cpo_Beta9_partial
XXXDAWNEPCVTSCGDSRAVVYPPPVAITFPGPILSSCPQESYVGTSEPLCIGGPYPAGGYLGYRGSVGTGCSYSSYSRQLNTYRYGSC
GPC

>Cpo_Beta10_partial
XXXAVVYPPPVAITFPGPILSSCPQESYVGTSEPLCIGGPYTAGGYLGYRGSVGTGYSYPSYSRQLNRYRYGGCGPC

>Cpo_Beta11
MSSYGQLISSRCYNPCEVTCPRPYADAWNEPCVTSCGDSRAVVYPPPVAITFPGPILSSCPQESYVGTSEPLCIGGLYPAGGYLGYRGS
VGTGCSYPSYSRQLNRYRYGGCGPC

>Cpo_Beta12
MSSYGQLISSRCYNPCEVTCPRPYADAWNEPCVTSCGDSRAVVYPPPVAITFPGPILSSCPQESYVGTSEPLCIGGPYPAGGYLGYRGS
VGTGYSYPSYSRQLNRYRYGGCGPC

>Cpo_Beta13
MSSYGQLISSRCYNPCEVTCPRPYADAWNEPCVTSCGDSRAVVYPPPVAITFPGPILSSCPQESYVGTSEPLCIGGPYPAGGYLGYRGS
VGTGYSYPSYSRQLNRYRYGGCRPC

>Cpo_Beta14
MSLYRQLLSSRCSNPCEVTCPQPYADAWNQPCVTSCGDSRAVVYPPPVVITFPGPILSSCPQESYVGSSAPISIGSSFGYGGSFTYGGS
LSYGGSFSTGSTYPCYSQRVKRYRYRSCGPCQTQKEFTCTRNTQETECKIQAQGLADDCEKC

>Cpo_Beta15
MSQSLSSRCLPPCSDICPKPCADAWNWPCVTSCGDSRAVVYPPPVVVHFPGPILASCPQESIVGTVEPRFPSNTGPYYPVGSGSGYGSG
GGFGFGSGYGSGSSYGFGSGYGFGSGYGSGSCYRSSRRYRKFSSGSCGPC

>Cpo_Beta16
MSFNRQLLSSRCFNPCEVTCPQPYANAWNEPCVTSCGDSRAVVYPPPVVVTFPGPILASCPQESYVGTSEPLQIGGSFLSGGSAGSGSS
LGCLSPYYSQRYNKYRYGNCGSC

>Cpo_Beta17
MSFNRQLLSSRCFNPCEVTCPQPYANAWNEPCVTSCGDSRAVVYPPPVVVTFPGPILASCPQESYVGTSEPLQIGGSFLSGGSAGSGSS
LGCLSPYYSQRYNKYRYGNCGSC

>Cpo_Beta18
MSTSGALCCYPPQPPCEVTCPRPYADAWNEPCVKSCGDSRAVVHPPPVVVTFPGPILASCPQESYVGTSLPQLSGSLSGSGGFIGSGGG
YGGSLGYRGSLGYGSSLGYGGSLGYGGSLGYGGSLGYGGPFCGLGGPICGYGSYGGSYSSGLSSYGGGYSSPFCSRRYSKYRY
GSCGPC

>Cpo_Beta19
MSCSENPCNDPCSTPCEAKCPKPQGITSNEPCVIACEDTRVIIYPPPVVVTFPGPILTTCPQETLVASTVTLAESSDDVTLAESPAMLP
SVPEVTRRSVPCDEICPPCIIPRPMPCYLPNYSYTFSTQWTHPCNRSGFKKYKSS

>Cpo_Beta20
MSQSLSSRCLPPCSDICPRPCVDAWNWPCVTSCGDSRAVVHPPPVVVHFPGPILASCPQESIVGTVEPRLLSNTGPYYSVGSGSGYGSG
GSYGIGSSYGSGGGYRIGSGYGSVGGYGMGSGYGSGGGYGMGSGYGYGSGYGPGFGCGFGSGYGSGGGYGFGGGYRSGSCYGSVSSRRY
RRYSSGSCGPC

>Cpo_Beta21
MALSSRCCPSVICPKPCVDACNWPCVTSCGDSKAVVYAPPVIVHFPGPILASCPQESIVGTVLPNPMRGGVGPYTSGSFGSGSSYGSGA
SYGSSSGFRSGSGFGFSDGYGLGGGYGSGGGYGSGGGYGFGCGYGSDGGYGFGGGYRSSSCYGSVSSRRRRRYSSAGCGPC

>Cpo_Beta22_partial
MGSYGPLVSSGCYNPCEVACPEPCVQACNQPCVTSCGDSRAVVYAPPVIVTFPGPILSTCPQESLVGTVLPYESGRPIPMRGSSYGGGS
SFGSGGFTGGXXX

>Cpo_Beta23_partial
MGSYGPLVSSGCYNPCEVACPEPCVQACNQPCVTSCGDSRAVVYAPPVIVTFPGPILSTCPQESLVGTVLPYESGRPIPMRGSSYGGGS
SFGSGGFTGGXXX

>Cpo_Beta24
MGSYGPLVSSGCYNPCEVACPEPCVRACNQPCVTSCGDSRAVVYAPPVIVTFPGPILSTCPQESLVGTVLPYESGRPIPMRGSSYGGGS
SFGSGGFTGGSYGGGFSGGLSSGGGSYGGGYGGLSGGGGSYGGGSYGGSSGGGGFSSGGFGYGGLCGGGGSFGGGSFSGGSSGGGYRRS
CGYRRSYTSGRATFGSSGGSRRGSIGPC

>Cpo_Beta25_partial
MSCGQMLSSRCLPPCEMMCPEPYATACNYPCTTSFGDSKAVVFAPPVIMTFPGPILATCPQESVVGAAEPYPVGGFPGGPYEGSGGSYG
GSYGNYGGSYGGSSGFGGSYGTSRRSYGGSSGTGGSSGATYGSSGHSYGISGGSHSSGTSYGGSGGSRGGSGGIGGSSGSSGGSYGMSG
GSRGSSTSYGGTGGSRGGSGRIGVSSGSSGGSYGMSGGSHGSTTSYGGTGGSRGGSGGIGVSSGSSGGSYGTSGGSHESAXXXGGSGGI
GVSSGSSGGSYGNSGGSHESETSYGGSGGSHEGSGGSHGVSGAIKGSSGSSGCFYGMSGDSYGFGDSCEGSGGSCDVEGSGIFRSSFFS
RYSPGNFRNTFPFTRFSYQRQFGNNEFF

>Cpo_Beta26
MSCNTDHCTEGRPSPCEVKCPQPIVTSTNEACVVSCGDSRVIIYPPPVIVTFPGPILSTCPQESLVGAAVPCESGVPQSATTVPLTSEI
GGSSGFSVPLRSEIGGNSGFSVPLRSEIMGNSGFSAERLYLNREPQQPSTYTYSFTSQWRHPCNRPGWNRYQSSYMKKEEPEEEEKPKE

Supplementary Figure S2. Amino acid sequences of proteins encoded by EDC genes of the saltwater crocodile (Cpo, *C. porosus*). **(A)** Amino acid sequences of EDC proteins apart from corneous beta proteins (CBPs). **(B)** Amino acid sequences of CBPs, also called beta-keratins. Several amino acid residues are highlighted to indicate the peculiar amino acid compositions of SEDC and SFTP proteins (Fig. 5). Cysteine (C) is highlighted in yellow, proline (P) in green, lysine (K) in cyan and glutamine (Q) in grey. Serine (S) and glycine (G) are shown with bold fonts colored red and orange, respectively. Three X´s indicate an unknown numbers of amino acid residues due to gaps in the corresponding gene sequences. Among S100A proteins, only those encoded by genes flanking *PGLYRP3* and *Scfn* are shown here.

```
TATA box                        <             non-coding exon 1            >         intron
tataaaagctctcccatcccagtcctcctcattcagctcctcgtctctctccgcatttctccttgctgctgaaagcggtaagtctgagtaaact
ctttctccttaggattcagagcagcagctttcttagagtatcttttcgagaacagggtatgaaacatatcagtgggtgttatagaggggctttg
cctgagcccaaattgtggctgcctggctggtaacaccaaagacctgcacaggttgcactgagaatggggggaatattcaaccaaaggcacaaag
ctgattgatggtaataaagtcactgggcagatggaaaaaaagttcccaaagtccttgctggtgacatgctgggagaagacacagtgtcttcaga
gtatttctgacctctctgaaaccaggtgcatccaccttagaagttcaaaggaaaacagtctgctcaaggcccctcagaaacagtcctggggtcc
ctctcatccactctctctttcttgcagctttgcctacacaactcaaagatgtgctctcgcagatcttgccacgaccacggatcctcatcccatg
                                                M  C  S  R  R  S  C  H  D  H  G  S  S  S  H
gatgccatggacatgaatcgtcctgccacggctctagctcctccattccctgcatcatcgagaaacctgtgcccgtgtgtcccatgcaaccctg
G  C  H  G  H  E  S  S  C  H  G  S  S  S  S  I  P  C  I  I  E  K  P  V  P  V  C  P  M  Q  P  C
ctgccccctgtgcagcaatgctgcccacccgtgcaaaagtgctgccccccgtgcagcaatggtgcccacccatgcaaaagtgctgcccacct
  C  P  P  V  Q  Q  C  C  P  P  V  Q  K  C  C  P  P  V  Q  Q  W  C  P  P  M  Q  K  C  C  P  P
gtgaagtgctgtcagcagaccaagcagtgctgcaagttccctccacagtgccccaagtaagagccacagccatgaggaggacgccaagagctgc
 V  K  C  C  Q  Q  T  K  Q  C  C  K  F  P  P  Q  C  P  K  -
ctgctccattcatctccttgggatggtggcttcttggctgaaagcgtctttgtctgcattcaccatctgtcatttgtgttctcttcttcgtttt
atgttcatcactaaatgcctgtaacatgctgggcggtttcatccctgcatggagtgatatatttttacaagacataatgtgtggacttctgttt
gctatgtgtgtatcagagtctttctcctacctcatttcctttttcagtctcaattttgagatgcaaatacaataaagctcctctttcctgaaag
catcacactctggggaattatttacttttgactctggttgtttctgtcatctttctcagtcggtgtgtccttagactctagagtttaattcacg
```

Supplementary Figure S3. Identification of the coding sequence (EDCH6) in a transcript, that was predicted as non-coding RNA (ncRNA) in Alligator mississippiensis Annotation Release 102. The nucleotide sequence of gene *LOC106738316* [Alligator mississippiensis (American alligator)] (see also Figure 3A), predicted to be transcribed as long non-coding RNA (lncRNA) (XR_001373451.2) (blue fonts), is shown. tBLAST analysis with EDC-encoded proteins led to the identification of the coding sequence (yellow shading). The encoded amino acid sequence, corresponding to EDCH6, is shown underneath the nucleotide sequence. An amino acid sequence alignment with other EDCH proteins (Figure 6) demonstrated high sequence similarity to other members of this proteins family. The position of the TATA box (green), consensus splice signals (underlined) at the borders of the intron, and a putative poly-adenylation signal (double underline) are indicated.

>Ami_EDP1

```
MSYYGQQCKQRC
LPLPICQDQLPV
KCPPMCPQQWTP
QYSTKSYSGYDG
YCESSSLQCMEP
-CGPKGWL----
KCRPQCEQVYVP
PCPPPCVT----
KCPQQCVTQCVT
KCPPPCV-----
KCPPPCV-----
KCPQPCVTQCIT
KCPPPCVT----
KCPQQCVT----
KCPQQCVT----
KCPPPCVT----
KCPQQXVT----
KCPPPCVT----
KCPQQCVT----
KCPPPCVT----
KCPQQCVT----
KCPQQCVT----
KCPQQCVT----
KGQSSKVKISSN
NKKYCSASKWF-
```

>Ami_EDPE

```
-------MSSHQMQCK-QKTTLPPPL--CKGPPNQDQD-----------PVPLPEPVPLPAPIPDPGQGKTP
DIKIPECPPQQQQQCKLPP-IIPPCPPPCKEPPVPEPIPFPEPGPCPEKPVPLPAPMPEP------GKGKTP
DIKIPKCPPQQQQQCKEPPVIIPPCPPPCKEPPVPEPMPFPEPGPCPEKPVPLPLPTPLPAPMPEPGQGKTP
DIKIPECP-QQQQQCKLPP-IIPPCPPPCKEPPVPEPIPFPEPGPCPEKPLPLPTPIPDP------GQGKTP
DIKIPECPPQQQQQCKLPP-IIPPCPPPCKEPPVPEPMPFPEPEPCPEKPVPLPLPTPLPAPMPDPGQGKTP
DIKIPECPPQQQQQCKEPPVVIPPCPPPCKEPPV--------------------------------------
KCPPPCPPIQQQQQKQPCQWPPQQK-----------------------------------------------
```

>Ami_Lor

```
MSSTQQKTACQEIPQQSRGLQG-SSCHGGGSSVSSG
NGIGGASSCCGGSSGQNICVSG-SS-GSSCCTGGSG
YGIGGGFNYGGGSSGQKIGVTGGSSSGXXXXXXSSG
-QTIVGSGSGGSDYGIGGGSS--------CGGVSSG
HKTIVGSGSGGSGYGISGGSSYGTGGGSSSGGVSSG
HKTIVGSGSGGSSYGIGGGSS--------SGGVSSG
HKTIVGSGSGGSGYGTGGGSSYGTGGGSSSAGVSSG
HKTIVGSGSGGSGYGIGGGSSYGIGGGSSCAGGSSG
QTISIGSCSGSGGSSGISIGGPGIGGGLSSGG--SG
RSSSKLIINSRGSGGSCSISG-----SSSSGSGGSL
QSVPQHQTKQPCQWPPQQK-----------------
```

Supplementary Figure S4. Crocodilian EDC proteins contain sequence repeats. The amino acid sequences of three EDC proteins of the American alligator (*Alligator mississippiensis*, Ami) are shown whereby repetitive sequences are aligned. Dashes were introduced to optimize the alignment of repeats. X, unknown amino acid residue(s) due to gaps in the genome sequence assembly. Amino acid residues are color-coded according to Figure 5.

A

Amino-terminal sequence motif

```
Alligator EDPE     MSSH--QMQCKQKTTLPP
Alligator EDP3     MNL-----Q-KQEKQVPV
Alligator EDPCV    MSF---QHQCKQPCL-PP
Alligator EDYM1    MSYYGYQL--KQQCYVPP
Alligator EDP2     MSSRQNQQQCKQVPTLPP
Alligator EDQA     MYYP-QQHQCKQPCLPPP
Alligator EDDML    MAFPNQQQY-KQPCLPPL
Alligator EDPQ1    MSYSD-QQQCKQVVCPPP
  Chicken EDQCM    MSYY---EQCKQPCL-PP
  Chicken EDPE     M-------QCKQEVTLPP
  Chicken EDYM1    MSYWY---QYKQQCFIPS
  Chicken EDP3     MSSH---QQ-KQQQQIPA
    Human Lor      MSY-----QKKQPTPQPP
    Human PRR9     MSFS--EQQCKQPCVPPP
    Human SPRR1A   MNS---QQQ-KQPCTPPP
    Human SPRR1B   MSS---QQQ-KQPCTPPP
    Human SPRR2A   MSY--QQQQCKQPCQPPP
    Human SPRR2G   MSY--QQQQCKQPCQPPP
```

B

Carboxy-terminal sequence motif

```
Alligator Lor      HQT-KQPCQWPP---QQK*
Alligator EDQL     QQT-KQPIHWPPQQQHQK*
Alligator EDPE     QQQQKQPCQWPP---QQK*
  Chicken Lor1     QQT--QPISWPPQT-KHK*
  Chicken EDGH     QQI-KQSSQWPPS--QKK*
  Chicken EDPE     QQV-KQPSPWPLT---QK*
  Chicken EDQL     QQI-KQPVQWPT--QQQK*
    Human Lor      QQ--KQAPTWPS-----K*
    Human Ivl      QQ--KQEVQWPP---KHK*
```

Supplementary Figure S5. Crocodilian epidermal differentiation proteins contain evolutionarily conserved amino acid sequence motifs at their amino and carboxy-termini. Alignments of amino acid sequence motifs present at the amino-terminus **(A)** and carboxy-terminus **(B)** of proteins encoded by genes of the EDC in the alligator (*Alligator mississippiensis*), chicken, and human. Note that the motifs are conserved in some but not all proteins encoded in the EDC of crocodilians and other amniotes. The sequence motifs of human and chicken proteins were reported and discussed in detail previously (Strasser et al., 2014). Characteristic amino acid residues are highlighted by color-shading. Dashes were introduced to optimize the alignment. Full names of EDC-encoded proteins of sauropsids are listed in Suppl. Table S1. Ivl, involucrin; Lor, loricrin; PRR9, proline rich 9; SPRR, small proline rich protein. *, end of the protein.

A

```
Cmo_EDAA8(skin)  MFDSLDAIEDLCYQGQYDCWDPCYRRPYWYGCWDPCTYQRPYNYGNCYGYGGLYRLGGCYPYSSRWG-----------
Cpo_EDAA8        MFDSLDAIEDLCYQGQYDCWDPCYRRPYWYGCWDPCTYRRPYNYGNCYGYGGLYRLGGCYPYSSRWGRKYSYGNCWPC
```

B

```
Cmo_EDCH10(skin) MCSRGSCHDHGSSSHGCHGHESSCHGSSSSVNCIIEKPVPICPVPQCCPPVQQCCPPVQ-------------------------------
Cpo_EDCH10       MCSRGSCHDHGSSSHGCHGHESSCHSSSSSVNCIIEKPVPICPMPQCCPPVPQCCPPVQQCCPPVQQCCPPVQCCQQSQQCCKIPPQCPK
```

C

```
Cmo_EDWM(skin)   MICSSGRESYFNLNSTWYDPSGSWLENHRIPLRYADDSCCGGCNPDVRGVGGHNYRPYWYRRSVC--------------------------
Cpo_EDWM         MICSSGRESYFNLNSTWYDPSGSWLENHRIPLRYADDSCCGGCNPDVRGVGGHNYRPYWYRRSVCSEAERGSSSGYCGSEDSGCARRPTLG

Cmo_EDWM(skin)   -------------------------------------------------------------------------------------------
Cpo_EDWM         YSEGCDCYRRRPDCCNGECSSHEFGRRPTYHYAADVYLANERLACSDGCHGSSGGFYGSSGGCHRRRRCGEPCHGSGSYGFSRGCHGRCRS

Cmo_EDWM(skin)   -----------------------------------------------------------------------------------------
Cpo_EDWM         VCGEPCHDSGSSSHLLPVCVKPEPCIPRCPPKQKYVRSTQSCCIPVQSYCTPVKACCPPIQAYCPPVGKYSSGGQQCKQTSKLPTLKAK
```

Supplementary Figure S6. Amino acid sequences encoded by mRNAs in the skin of Morelet's crocodile are highly similar to EDC proteins of the saltwater crocodile. RNA from the skin of Morelet's crocodile (*Crocodylus moreletii*, Cmo) was reverse-transcribed and subjected to PCRs with intron-spanning primers (Suppl. Table S4). The nucleotide sequences of the PCR products (submitted to GenBank) were translated and the amino acid sequences were aligned to the sequences of proteins encoded in the EDC of the saltwater crocodile (*Crocodylus porosus*, Cpo). The amino acid sequence alignments of EDAA8 **(A)**, EDCH10 **(B)**, and EDWM **(C)** are shown. Red fonts indicate identical amino acid residues. As the antisense primers were designed to anneal within the coding region, the amino acid sequences of Morelet's crocodile proteins could predicted for the amino-terminus while the region encoding the carboxy-terminus (indicated by dashes) was not amplified.

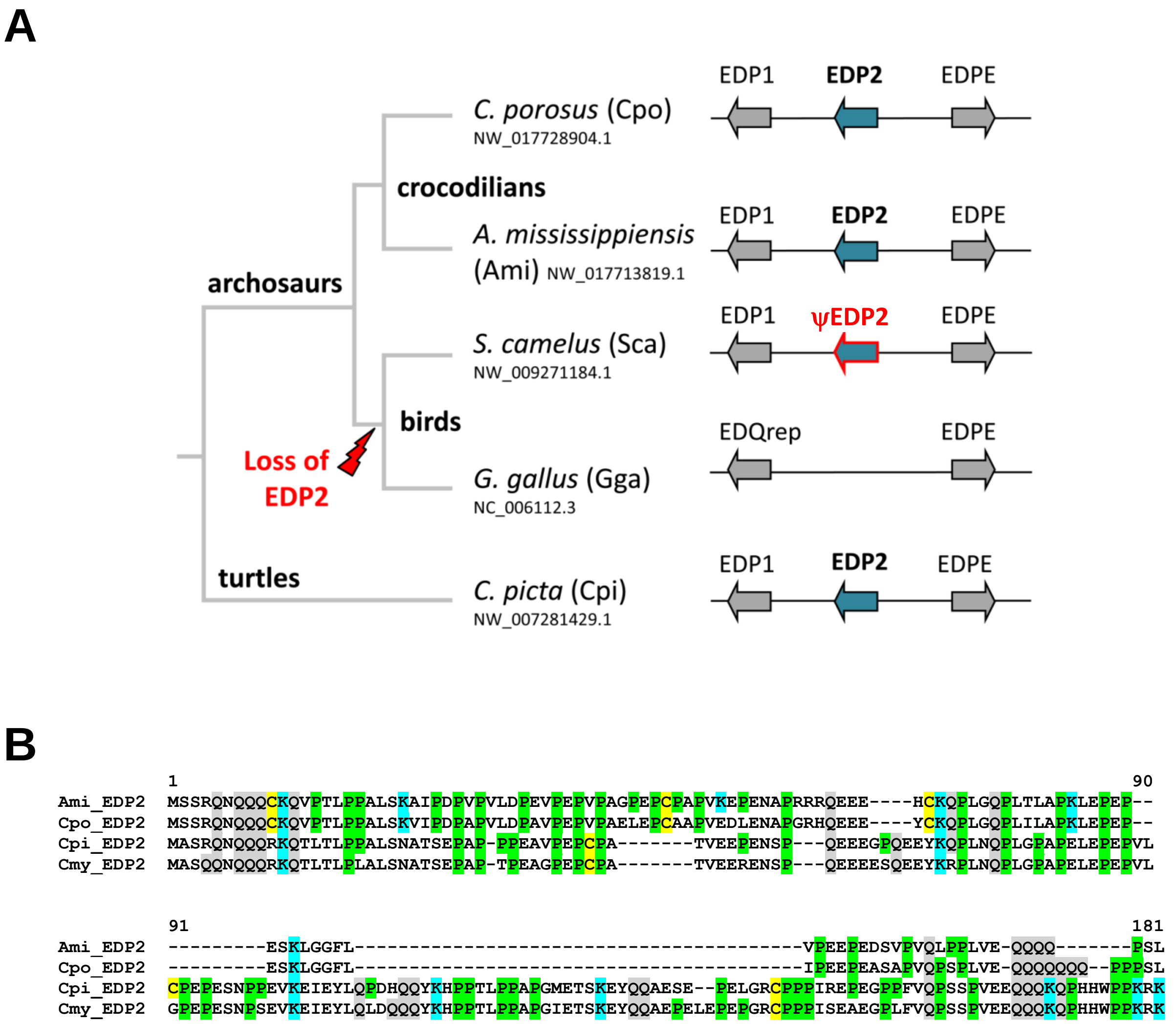

Supplementary Figure S7. The *EDP2* gene is conserved in crocodilians but not in birds. (A) Schematic phylogenetic tree of the *EDP2* locus with turtles as an outgroup to archosaurs represented by two bird and two crocodilian species. The gene locus is schematically depicted for each species with arrows representing genes. Note that an *EDP2* gene remnant (pseudogene, ψ) inactivated by mutations is present in the EDC of the ostrich (*Struthio camelus*). **(B)** Amino acid sequence alignment of EDP2 proteins of crocodilians (Ami, Cpo) and turtles (Cpi, Cmy). Species names are abbreviated as in panel **A**; Cmy, *Chelonia mydas*. Amino acid residues involved in protein cross-linking (C-C, Q-K) and the highly abundant proline residues are highlighted. Numbers indicate positions of the alignment.

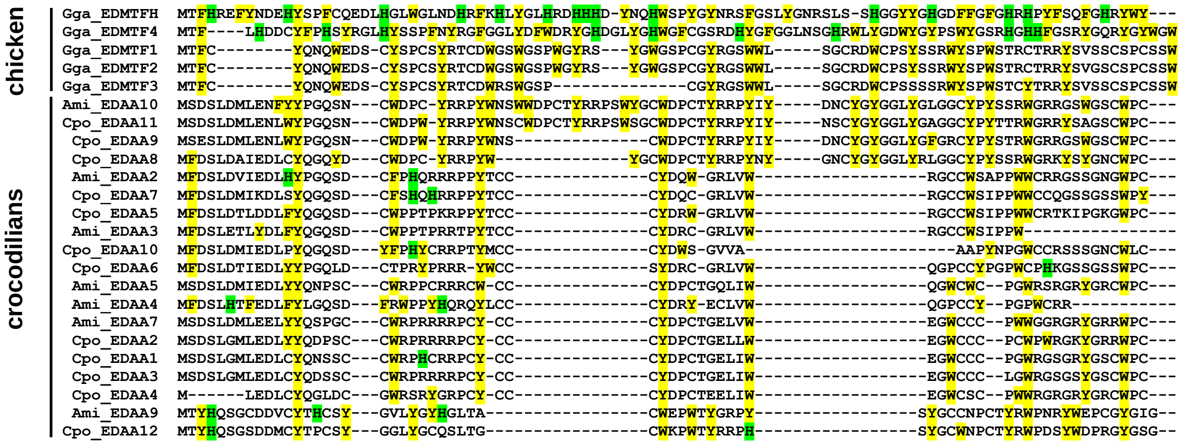

Supplementary Figure S8. Alignment of amino acid sequences of chicken EDMTFs and crocodilian EDAAs. Chicken *EDMTF* (*Epidermal Differentiation protein starting with an MTF motif*) and crocodilian *EDAA* (*Epidermal Differentiation protein rich in Aromatic Amino acids*) genes are located at syntenic positions in the EDC (Fig. 2). The amino acid sequences of the encoded proteins were aligned. Histidine residues (H) are highlighted by green shading, and other aromatic amino acid residues (F, Y, W) are highlighted by yellow shading. Note that chicken EDMTFH corresponds to a protein identified in feathers and originally termed histidine-rich protein (HRP); of note, its high histidine content is not conserved among birds (for details and further references see Alibardi et al., 2016). Gga, chicken (*Gallus gallus*); Ami, American alligator (*Alligator mississippiensis*); Cpo, saltwater crocodile (*Crocodylus porosus*).

A

```
               1                                                                                                  100
Gga_PGLYRP3    MMLRLAVLFSALCAASCQLACPPIVSPAKWGSRPAKCASPLSKVPPGNVVIIHTAGSACHTQPECSELLRNIQVFHRDMKEWCDVSYNFLIGEDGNVYEG
Cpo_PGLYRP3    MMLRLAVLFSALCAASCQLACPPIVSPAKWGSRPAKCASPLSKVPPGNVVIIHTAGSACHTQPECSELLRNIQVFHRDMKEWCDISYNFLIGEDGNVYEG
Ami_PGLYRP3    MMLRLAVLFSALCASSCQLACPPIISPAKWGSRPAKCAAPLSKVPPGNVIIIHTSGSACHTQPECSELLRNIQVFHRDMKEWCDISYNFLIGEDGNVYEG

               101                                                                           186
Gga_PGLYRP3    RGWLLEGAHTYGYNDLSLGIAFIGDFTERSPNEAAWKALKNLLAYAIQSGYLASDYLLMAHSDVSNTVSPGKLIRETIKMWPHYKH
Cpo_PGLYRP3    RGWLLEGAHTYGYNDLSLGIAFIGNFTERSPNEAAWKALKNLLAYAVQSGYLASDYLLMAHSDVSNTISPGKLIRETIKLWPHYKH
Ami_PGLYRP3    RGWLLEGAHTYGYNDLSLGIAFIGNFTERSPNEAAWKTLKNLLTYAVQSGYLASDYLLMAHSDVSNTISPGKLIRETIKMWPHYKH
```

B

```
               1                                                                                                 99
Gga_EDCRPL1    MCSCCSGCHGTRSVQPICYVQPVCCEPVYIQRSLGSCCQPCGSCCGSCCG----RSRSRPRVVIQRWPMPVCCPPLQYSAPM-------QQCCSPLKKY
Ami_EDCRPL1    MCSCCSGCHGTRSVQPICYVQPVCCEPVYIHRSSGSCCQPCGSCCGSCCR----GSRACPWVVIQRRPMPVCCPPLQYSAPM-------QQHCSPLKKC
Cpo_EDCRPL1    MCSCCSGCHGTGSVQPICYVQPVCCELVYIQRSSGSCCQPCGSCCGSCCG----GSRSCPRVVMQRCPMLVCCPPLQYLAPM-------QQCWLPLKKC
Ami_EDCRPL2    MCSCCSGCHGT---ETICYVQPVCCEPVYIHRSSGSCCQPCGSCCGSCCGSCCWGLRSCPQVVIQRRPMPVCCPPLQYSSPM-------QQCCSPLKKC
Ami_EDCRPL3    MCSCCSGCHGTD--ETICYVQPVCCEPVYIQRSLESCCQPCGSCCGSCCG----GSRSCPRVVIQRRPMPVCCPPLQYSAPM-------HQCCLPLKKC
Ami_EDCRPL4    MCSCCSGCHGTES------VQPICCEPVYIQRSSGSCCQPCGSCCGSCCG----GSRPFPRVVIQRRPMPVCCPPLQYSAPMRKYSAPMQQCCPPLKKC
```

C

```
           1                                                                                                  100
Gga_CRNN   MTQLQGNIEGIISAFNAYAKKDGGCITLSKGELRQLIQQEFADVLVKPHDLQTIDQVLQRLDAESEDRIDFDEFLVLVFQVAKACHKELNPCQPSGDGQS
Cpo_CRNN   MTQLQGNIEGIISAFNAYAKEDGGCITLSKGELRQLIQQEFPDVLVKPHDLQTIDQVLQHLDAESEDRIDFDEFLVLVFQVAKACHKELNPCQPSGDGQS
Ami_CRNN   MTQLQGNIEGIVSAFNAYAKEDGGCITLSKGELRQLIQQEFADVLVKPHDLQTIDQVLQRLDVEREDRIDFDEFLVLVFQVAKACHKKLSPCQPSGDGQG

           101                                                                                                200
Gga_CRNN   SASQGDASRGQAQKAEHDPGHHHVQEPQAPKQGQKQPEAPEQDPSRPQAPETPTAEGDLSHRHIQDPEVS--------QGDGSREAQATETPEHDSICRQ
Cpo_CRNN   SASQGDASRGQAQRAEEDPGRRHIQEPQALEQGQKQPEAPEQDASRPQAPETPTAEGDLSHRHTQDPEVS--------QGDGNREAQAAETPEHDSVRRQ
Ami_CRNN   SAAQGDASRDQAQRADQ-------------EQGQKQPDAPEQDPTRPQAPETRTAEGDLSRRHTQDPEVSGTQDPEVSQGDGNHEAQAAKTPEHDSIRRQ

           201                                                                                                300
Gga_CRNN   GQEPEQDPSHHRTQKQDPNREGQAPQVPQQDVKHEALEPGAPEQAPNRHPVLQPSVSERDLDHCQSSASERGLDRHPALQP-------------------
Cpo_CRNN   GQELEQDPSHHRAQEQDPNREGQDPQVPQQDVTHEALESGALEQAPNRHP---------------------------ALQS-------------------
Ami_CRNN   GQEPEQDPSHHRAQEQDPNREGQDPQVPQQDVKHEALESGAPEQAPNRHPVLQPSVSERDLDHSHSSASERDLDHHPTLQPNTLETDLDRHPTLQPNTLE

           301                                                                                                400
Gga_CRNN   -----------STSETDLDRHQGLESEAPEQDLSPTAERESTHYEAHEPQAPEQESQESQPAEQDQNRHQSEEPETSEHTLCHQPQEAQPTEQDLTRETQ
Cpo_CRNN   -----------STSETDLDRHQGLQSEAPEQDLSPTAERESTHYEAHEPQAPEQGSQESQPAEQDQNRHQSEEPETLEHTLRHQPQEAQPTKQDLTQETQ
Ami_CRNN   TDLDRLPTLQPSTSETDLDHHQGLETEAPEQDLNSTTGRESTHNEAHELQAPEQESQESQPDEQDQNRHQSEEPEASEHTLHHQPQEAQPTEQDLTWETQ

           401                                                       459
Gga_CRNN   TPRVPEGDVSRGGTPFSPALQQDRGAQQDPREEALTAYRPYIYQCQKPPTFPYQWLPK-
Cpo_CRNN   TPRVPEGDVSRGGTPFSPALQQDRGAQQDPREEALTAYRPYIYQCQTPPTFPYQWLPKQ
Ami_CRNN   TPRVPEGDVSRGGTPFSPALQQDRGAQQDPREEALTAYRPYIYQCQKPPTFPYQWLPKQ
```

Supplementary Figure S9. Amino acid sequences alignments of proteins encoded by exemplary EDC genes of the Indian gharial. Preliminary results of a study of the EDC in the Indian gharial (*Gavialis gangeticus*, Gga) (Green et al. 2014) led to the identification of EDC genes on a single genome sequence scaffold (GenBank accession number NW_017728960.1). Three examplary genes, i.e. **(A)** *PGLYRP3* (nucleotide positions of the coding sequence: 2217140-2220459, start - stop), **(B)** *EDCRPL1* (nucleotide positions of the coding sequence: 2268030-2267764), and **(C)** *CRNN* (nucleotide positions of the coding sequence: 3242043-3244752) were analysed further. The amino acid sequences of the encoded proteins were aligned with the sequences of orthologs of the saltwater crocodile (*Crocodylus porosus*, Cpo) and the American alligator (*Alligator mississippiensis*, Ami).

Supplementary Table S1. Tentative abbreviations and full names of EDC genes in crocodilians.

Gene name abbreviation	Full gene name
Crnn	Cornulin
EDAA1	Epidermal Differentiation protein rich in Aromatic Amino acids 1
EDAA2	Epidermal Differentiation protein rich in Aromatic Amino acids 2
EDAA3	Epidermal Differentiation protein rich in Aromatic Amino acids 3
EDAA4	Epidermal Differentiation protein rich in Aromatic Amino acids 4
EDAA5	Epidermal Differentiation protein rich in Aromatic Amino acids 5
EDAA6	Epidermal Differentiation protein rich in Aromatic Amino acids 6
EDAA7	Epidermal Differentiation protein rich in Aromatic Amino acids 7
EDAA8	Epidermal Differentiation protein rich in Aromatic Amino acids 8
EDAA9	Epidermal Differentiation protein rich in Aromatic Amino acids 9
EDAA10	Epidermal Differentiation protein rich in Aromatic Amino acids 10
EDCH1	Epidermal Differentiation protein containing Cysteine Histidine motifs 1
EDCH2	Epidermal Differentiation protein containing Cysteine Histidine motifs 2
EDCH3	Epidermal Differentiation protein containing Cysteine Histidine motifs 3
EDCH4	Epidermal Differentiation protein containing Cysteine Histidine motifs 4
EDCH5	Epidermal Differentiation protein containing Cysteine Histidine motifs 5
EDCH6	Epidermal Differentiation protein containing Cysteine Histidine motifs 6
EDCH7	Epidermal Differentiation protein containing Cysteine Histidine motifs 7
EDCH8	Epidermal Differentiation protein containing Cysteine Histidine motifs 8
EDCH9	Epidermal Differentiation protein containing Cysteine Histidine motifs 9
EDCH10	Epidermal Differentiation protein containing Cysteine Histidine motifs 10
EDCH11	Epidermal Differentiation protein containing Cysteine Histidine motifs 11
EDCH12	Epidermal Differentiation protein containing Cysteine Histidine motifs 12
EDCH13	Epidermal Differentiation protein containing Cysteine Histidine motifs 13
EDCH14	Epidermal Differentiation protein containing Cysteine Histidine motifs 14
EDCH15	Epidermal Differentiation protein containing Cysteine Histidine motifs 15
EDCH16	Epidermal Differentiation protein containing Cysteine Histidine motifs 16
EDCH17	Epidermal Differentiation protein containing Cysteine Histidine motifs 17
EDCH18	Epidermal Differentiation protein containing Cysteine Histidine motifs 18
EDCH19	Epidermal Differentiation protein containing Cysteine Histidine motifs 19
EDCH20	Epidermal Differentiation protein containing Cysteine Histidine motifs 20
EDCH21	Epidermal Differentiation protein containing Cysteine Histidine motifs 21
EDCH22	Epidermal Differentiation protein containing Cysteine Histidine motifs 22
EDCH23	Epidermal Differentiation protein containing Cysteine Histidine motifs 23
EDCH24	Epidermal Differentiation protein containing Cysteine Histidine motifs 24
EDCH25	Epidermal Differentiation protein containing Cysteine Histidine motifs 25
EDDML	Epidermal Differentiation protein containing DPCC Motifs-Like
EDKM	Epidermal Differentiation protein containing a KKLIQQ Motif
EDP1	Epidermal Differentiation protein rich in Proline 1
EDP2	Epidermal Differentiation protein rich in Proline 2
EDP3	Epidermal Differentiation protein rich in Proline 3
EDPCV	Epidermal Differentiation protein rich in Proline (P), Cysteine (C) and Valine (V)
EDPCQ	Epidermal Differentiation protein rich in Proline (P), Cysteine (C) and glutamine (Q)
EDPE	Epidermal Differentiation protein rich in Proline and glutamic acid (E)
EDPL	Epidermal Differentiation Proline-rich protein, close to Loricrin
EDPQ1	Epidermal Differentiation protein rich in Proline and glutamine (Q) 1
EDPQ2	Epidermal Differentiation protein rich in Proline and glutamine (Q) 2
EDPQ3	Epidermal Differentiation protein rich in Proline and glutamine (Q) 3
EDPQ4	Epidermal Differentiation protein rich in Proline and glutamine (Q) 4
EDRYA	Epidermal Differentiation protein containing a RYA terminus
EDQA	Epidermal Differentiation protein containing a QA repeats
EDQL	Epidermal Differentiation protein rich in glutamine (Q), close to Loricrin
EDQM1	Epidermal Differentiation protein containing a glutamine (Q) Motif 1
EDQM2	Epidermal Differentiation protein containing a glutamine (Q) Motif 2
EDCRPL1	Epidermal Differentiation Cysteine-Rich Protein-Like 1
EDCRPL2	Epidermal Differentiation Cysteine-Rich Protein-Like 2
EDCRPL3	Epidermal Differentiation Cysteine-Rich Protein-Like 3
EDCRPL4	Epidermal Differentiation Cysteine-Rich Protein-Like 4
EDWM	Epidermal Differentiation protein containing a WYDP Motif
EDYM1	Epidermal Differentiation protein containing Y Motif 1
Lor	Loricrin
PGLYRP3	Peptidoglycan recognition protein 3
Scfn	Scaffoldin

Note - corneous beta protein (CBP)/beta-keratin genes and S100A genes are not included here.

Supplementary Table S2. American alligator (*Alligator mississippiensis*) EDC genes.

Gene	Accession nr.	CDS start	CDS end	Expression confirmed by RNA-seq evidence*
S100A9	NW_017707560.1	225447	227360	yes
PGLYRP3	NW_017707560.1	247213	250774	yes
EDKM	NW_017707560.1	255033	255730	yes
EDQM1	NW_017707560.1	262085	261567	yes
EDQM2	NW_017707560.1	270990	270697	no
EDWM	NW_017707560.1	281501	280707	yes
EDRYA	NW_017707560.1	286040	285756	yes
EDCRPL1	NW_017707560.1	289596	289330	no
EDCRPL2	NW_017707560.1	293091	292822	yes
EDCRPL3	NW_017707560.1	296698	296438	yes
EDCRPL4	NW_017707560.1	299657	299926	no
EDCH1	NW_017707560.1	304044	303793	yes
EDCH2	NW_017707560.1	308118	307846	yes
EDCH3	NW_017707560.1	316450	316199	yes
EDCH4	NW_017707560.1	322806	322555	yes
EDCH5	NW_017707560.1	336059	335808	yes
EDCH6	NW_017707560.1	349392	349685	yes
EDCH7	NW_017707560.1	363655	363906	yes
EDCH8	NW_017707560.1	369579	369830	yes
EDCH9	NW_017707560.1	376669	376397	yes
EDCH10	NW_017707560.1	379595	379822	yes
EDCH11	NW_017707560.1	391159	390866	yes
EDCH12	NW_017707560.1	401481	401116	yes
EDCH13	NW_017707560.1	409349	409642	yes
EDCH14	NW_017708735.1	>1770	1579	n.a.
EDCH15	NW_017709850.1	<1	180	n.a.
EDCH16	NW_017709850.1	>4409	4230	n.a.
EDCH17	NW_017709094.1	<1	189	n.a.
EDCH18	NW_017709094.1	5274	5534	n.a.
EDCH19	NW_017709094.1	10871	10590	n.a.
EDCH20	NW_017709094.1	13791	14093	n.a.
EDCH21	NW_017709094.1	17255	16995	n.a.
EDCH22	NW_017709094.1	19846	20085	n.a.
EDCH23	NW_017709094.1	26675	26415	n.a.
EDCH24	NW_017709094.1	30403	30101	n.a.
EDCH25	NW_017709094.1	37836	37594	n.a.
EDQL	NW_017711636.1	97382	97627	yes
LOR	NW_017711636.1	88286	89428	yes
EDPL	NW_017711636.1	66111	66296	no
EDbeta1	NW_017711636.1	57371	57766	no
EDbeta2	NW_017711636.1	51593	51279	yes
EDYM1	NW_017711636.1	63281	63763	no
EDAA1	NW_017711636.1	31628	31865	yes
EDAA2	NW_017708699.1	19759	19956	n.a.
EDAA3	NW_017708699.1	37757	37915	n.a.
EDAA4	NW_017708699.1	41629	41462	n.a.
EDAA5	NW_017708699.1	47513	47322	n.a.
EDAA6	NW_017708931.1	44	<1	n.a.
EDAA7	NW_017712395.1	2453	2262	n.a.

Supplementary Table S2. American alligator (*Alligator mississippiensis*) EDC genes (continued).

Gene	Accession nr.	CDS start	CDS end	Expression confirmed by RNA-seq evidence*
EDAA8	NW_017710154.1	759	481	yes
EDAA9	NW_017712382.1	41428	41231	no
EDAA10	NW_017712382.1	45444	45722	yes
Beta1	NW_017713118.1	13134	12730	yes
Beta2	NW_017713118.1	37872	37513	yes
Beta3	NW_017713118.1	54569	54063	no
Beta4	NW_017713118.1	63602	63979	no
Beta5	NW_017709396.1	83804	84220	yes
Beta6	NW_017709396.1	66815	67336	no
Beta7	NW_017709396.1	56602	56946	yes
Beta8	NW_017709396.1	51448	51792	yes
Beta9	NW_017709396.1	44067	44411	yes
Beta10	NW_017709396.1	36466	36810	yes
Beta11	NW_017709396.1	30006	30350	yes
Beta12	NW_017709396.1	62952	62608	yes
Beta13	NW_017710460.1	4869	5324	n.a.
Beta14	NW_017710607.1	593	255	yes
Beta15	NW_017713819.1	442399	443361	yes
Beta16	NW_017713819.1	422904	423338	yes
Beta17	NW_017713819.1	411860	412384	yes
Beta18	NW_017713819.1	409171	409554	yes
Beta19	NW_017713819.1	402867	402376	yes
Beta20	NW_017713819.1	37627	38013	no
Beta21	NW_017713819.1	40316	40840	yes
EDPCQ	NW_017713819.1	339407	340822	yes
EDP1	NW_017713819.1	332020	332874	yes
EDP2	NW_017713819.1	317856	318209	yes
EDPE	NW_017713819.1	307897	306749	yes
EDP3	NW_017713819.1	297508	297726	no
EDPQ1	NW_017713819.1	293018	292815	yes
EDPQ2	NW_017713819.1	283590	283384	no
EDPQ3	NW_017713819.1	274454	274660	yes
EDPQ4	NW_017713819.1	269429	269193	yes
EDPCV	NW_017713819.1	265095	265406	yes
EDDML	NW_017713819.1	255761	254523	no
EDQA	NW_017713819.1	247134	249220	yes
CRNN	NW_017713819.1	226344	223555	yes
SCFN	NW_017713819.1	213875	204788	no
S100A11	NW_017713819.1	200764	198379	yes

Notes - * "RNA-seq evidence" corresponds to the presence of RNA-seq peaks in the "Genomic regions, transcripts and products" view at the NCBI GenBank website (www.ncbi.nlm.nih.gov, 2017). For genes located on some scaffolds, this view was not available (n.a.).
EDBeta and Beta followed by a number refer to corneous beta protein (CBP)/beta-keratin genes. Among S100A genes only those flanking PGLYRP3 and SCFN are shown here. The symbols < and > indicate that ends of the coding sequence were not present on the scaffold. CDS, coding sequence.

Supplementary Table S3. Saltwater crocodile (*Crocodylus porosus*) EDC genes.

Gene	Accession nr.	CDS start	CDS end
S100A9	NW_017728904.1	<243744	245030
PGLYRP3	JRXG01058286.1	2179	<232
	JRXG01058285.1	>626	450
EDKM	JRXG01058284.1	952	252
EDQM1	JRXG01006413.1	5178	5645
EDQM2	NW_017728904.1	290589	290200
EDWM	NW_017728904.1	299694	298879
EDRYA	NW_017728904.1	305213	304929
EDCRPL1	NW_017728904.1	308751	308485
EDCH1	NW_017728904.1	318243	317992
EDCH2	NW_017728904.1	322239	321967
EDCH3	NW_017728904.1	328285	328034
EDCH4	NW_017728904.1	332130	331879
EDCH5	NW_017728904.1	335663	335391
EDCH6	NW_017728904.1	>339228	339166
EDCH7	JRXG01044411.1	2786	2514
EDCH8	JRXG01044411.1	>7080	7000
EDCH9	JRXG01044410.1	8965	>9174
EDCH10	JRXG01044410.1	6192	6464
EDCH11	JRXG01044410.1	3294	3058
EDCH12	JRXG01044410.1	575	808
EDCH13	JRXG01013561.1	<1	175
EDCH14	JRXG01039386.1	5323	5090
EDCH15	JRXG01099277.1	1279	1539
EDCH16	JRXG01099277.1	8174	7932
EDCH17	NW_017728904.1	344777	344499
EDQL	NW_017728904.1	353105	352866
LOR	NW_017728904.1	363815	362264
EDPL	NW_017728904.1	385663	385451
EDYM1	NW_017728904.1	388477	387995
EDbeta1	NW_017728904.1	394371	393976
EDbeta2	NW_017728904.1	400062	400376
EDAA1	JRXG01066409.1	1710	1901
EDAA2	JRXG01066410.1	3519	3328
EDAA3	JRXG01076834.1	4212	4397
EDAA4	JRXG01063821.1	2219	2046
EDAA5	JRXG01104627.1	4938	4741
EDAA6	JRXG01076832.1	17367	17561
EDAA7	JRXG01076833.1	5689	5492
EDAA8	JRXG01069781.1	2547	2783
EDAA9	JRXG01060709.1	13537	13301
EDAA10	JRXG01060709.1	4202	4387
EDAA11	JRXG01060708.1	17706	17428
EDAA12	JRXG01060708.1	22931	23128
EDAA13	JRXG01104928.1	2430	>2603
EDAA14	JRXG01076836.1	>15917	15705
EDAA15	JRXG01099001.1	128	<1
Beta1	NW_017728904.1	425383	425778

Supplementary Table S3. Saltwater crocodile (*Crocodylus porosus*) EDC genes (continued).

Gene	Accession nr.	CDS start	CDS end
Beta2	NW_017728904.1	430625	430230
Beta3	NW_017728904.1	454438	454079
Beta4	JRXG01074025.1	3021	2515
Beta5	JRXG01074025.1	16939	16616
Beta6	JRXG01074025.1	28647	28060
Beta7	JRXG01110871.1	14074	14595
Beta8	JRXG01110870.1	15546	>15716
Beta9	JRXG01110870.1	<1	271
Beta10	JRXG01110869.1	<1	225
Beta11	JRXG01004993.1	2781	2437
Beta12	JRXG01110864.1	1952	2296
Beta13	JRXG01110865.1	2994	3338
Beta14	JRXG01004994.1	5855	5400
Beta15	JRXG01086413.1	2155	1736
Beta16	JRXG01011795.1	3986	4324
Beta17	JRXG01011795.1	10908	11246
Beta18	JRXG01011795.1	17328	16774
Beta19	JRXG01011795.1	38823	38389
Beta20	NW_017728904.1	590427	590167
Beta21	NW_017728904.1	595099	595419
Beta22	NW_017728904.1	600837	<600541
Beta23	NW_017728904.1	605656	<605360
Beta24	NW_017728904.1	611827	611207
Beta25	NW_017728904.1	625170	623850
Beta26	NW_017728904.1	641121	640585
EDPCQ	NW_017728904.1	653561	652116
EDP1	NW_017728904.1	662837	661880
EDP2	NW_017728904.1	681392	681024
EDPE	NW_017728904.1	691240	692343
EDP3	NW_017728904.1	699606	699430
EDPQ1	NW_017728904.1	704196	704399
EDPQ2	NW_017728904.1	709497	709703
EDDML	NW_017728904.1	721051	722289
EDQA	NW_017728904.1	728952	727573
CRNN	NW_017728904.1	749469	752090
SCFN	NW_017728904.1	762021	770451
S100A11	NW_017728904.1	<776095	776844

Notes - EDBeta and Beta followed by a number refer to corneous beta protein (CBP)/beta-keratin genes. Among S100A genes only those flanking PGLYRP3 and SCFN are shown here. The symbols < and > indicate that ends of the coding sequence were not present on the scaffold. CDS, coding sequence.

Supplementary Table S4. Design of primers for RT-PCR analysis of Morelet's crocodile (*Crocodylus moreletii*) EDC genes.

Target gene	Exon in which primer anneals	Orientation of primer relative to gene	Scaffold (Accession number)	Annealing site (Nucleotide numbers)	Primer sequence (5'-3')
EDAA8	exon 1	sense	JRXG01069781.1	1531-1551	ACTTCTCTCTTCAGTGAGCCG
EDAA8	exon 2	anti-sense	JRXG01069781.1	2748-2768	GTTGCCATAGCTGTACTTGCG
EDCH10	exon 1	sense	JRXG01044410.1	5573-5593	CGCTTGTTCTTCCTGCTGATC
EDCH10	exon 2	anti-sense	JRXG01044410.1	6391-6410	CTGCACAGGTGGGCAGCATT
EDWM	exon 1	sense	NW_017728904.1	300146-300166	CTCACCTGTCCTCACTACTCA
EDWM	exon 2	anti-sense	NW_017728904.1	299479-299499	AGACCCGCGCTCTGCCTCAGA

Note - The primers were designed based on the genome sequence of *C. porosus* and were used to amplify cDNAs of *C. moreletii* .
